# Tiliacorinine as a Promising Candidate for Cholangiocarcinoma Therapy via Oxidative Stress Molecule Modulation: A Study Integrating Network Pharmacology, Molecular Docking and Molecular Dynamics Simulation

**DOI:** 10.3390/antiox14111273

**Published:** 2025-10-23

**Authors:** Tavisa Boonsit, Moragot Chatatikun, Suphasarang Sirirattanakul, Nawanwat C. Pattaranggoon, Imran Sama-ae, Fumitaka Kawakami, Motoki Imai, Pritsana Raungrut, Atthaphong Phongphithakchai, Aman Tedasen, Saowanee Maungchanburi

**Affiliations:** 1 School of Allied Health Sciences, Walailak University, Nakhon Si Thammarat 80161, Thailand; tavisa.bo@mail.wu.ac.th (T.B.); moragot.ch@wu.ac.th (M.C.); suphasarang.si@wu.ac.th (S.S.); imran.sa@wu.ac.th (I.S.-a.); 2 Research Excellence Center for Innovation and Health Products, Walailak University, Nakhon Si Thammarat 80161, Thailand; 3 Faculty of Medical Technology, Rangsit University, Pathum Thani 12000, Thailand; nawanwat.p@rsu.ac.th; 4 Center of Excellence Research for Melioidosis and Microorganisms (CERMM), Walailak University, Nakhon Si Thammarat 80161, Thailand; 5 Research Facility of Regenerative Medicine and Cell Design, School of Allied Health Sciences, Kitasato University, Sagamihara 252-0373, Japan; kawakami@kitasato-u.ac.jp (F.K.); imai-m@kitasato-u.ac.jp (M.I.); 6 Department of Regulatory Biochemistry, Kitasato University Graduate School of Medical Sciences, Sagamihara 252-0373, Japan; 7 Department of Molecular Diagnostics, School of Allied Health Sciences, Kitasato University, Sagamihara 252-0373, Japan; 8 Department of Biomedical Sciences and Biomedical Engineering, Faculty of Medicine, Prince of Songkla University, Songkhla 90110, Thailand; rpritsana@gmail.com; 9 Nephrology Unit, Division of Internal Medicine, Faculty of Medicine, Prince of Songkla University, Songkhla 90110, Thailand; ton331@hotmail.com

**Keywords:** tiliacorinine, cholangiocarcinoma, ADMET, network pharmacology, molecular docking, molecular dynamics simulation

## Abstract

Cholangiocarcinoma (CCA), an aggressive biliary tract cancer whose prevalence is rising, particularly in Thailand, is marked by elevated oxidative stress driven by chronic inflammation, parasitic infections, and dysregulated redox signaling. This study investigates the anticancer potential of tiliacorinine using a silico approach, including drug-likeness, ADMET, network pharmacology, molecular docking, and dynamics simulations. Tiliacorinine and 216 predicted targets were identified, with 79 overlapping CCA-related genes from GeneCards. GO and KEGG analyses revealed involvement in cell migration, membrane structure, kinase activity, and cancer-associated pathways. Network and PPI analyses identified ten key targets, including SRC, HIF1A, HSP90AA1, NFKB1, MTOR, MMP9, MMP2, PIK3CA, ICAM1, and MAPK1. Tiliacorinine showed the strongest affinity for MTOR (−10.78 kcal/mol, K_i_ = 12.62 nM), binding at the same site as known inhibitors with superior energy and specificity, supported by hydrogen bonding at ASP950 and hydrophobic interactions. Tiliacorinine also demonstrated strong binding to SRC, MMP9, and MAPK1. Molecular dynamics simulations revealed stable binding of tiliacorinine to MTOR, particularly at residues ASP950, TRP1086, and PHE1087. Comparative analysis with the MTOR–GDC-0980 complex confirmed consistent interaction patterns, reinforcing the structural stability and specificity of tiliacorinine. These results highlight its strong pharmacological potential and support its candidacy as a promising lead compound for cholangiocarcinoma therapy.

## 1. Introduction

Cancer constitutes a vast and diverse collection of diseases characterized by the unchecked proliferation of aberrant cells that invade adjacent tissues and disseminate to distant organs, a capacity that underlies the approximately 9.7 million cancer-related deaths out of nearly 20 million new cases reported worldwide in 2022 (GLOBOCAN 2022) [1]. Among these malignancies, cholangiocarcinoma (CCA) emerges as a formidable and heterogeneous biliary tract cancer, representing roughly 15 percent of primary liver tumors and 3 percent of gastrointestinal malignancies [2]. Several studies have shown a rising trend in CCA incidence across Western countries. Between 2000 and 2004, male mortality rates in Europe were approximately 2 per 100,000, increasing to roughly 3 per 100,000 during 2010–2014 [3,4,5,6]. Cholangiocarcinoma imposes a particularly heavy burden in Southeast Asia, with Thailand exhibiting the world’s highest incidence rate [7]. Anatomically, CCA is subdivided into intrahepatic (ICC) and extrahepatic (ECC) forms, the latter further classified as perihilar or distal based on its biliary location [8,9]. Significant risk factors include male sex, older age, non-alcoholic fatty liver disease, obesity, and diabetes mellitus, with chronic infection by the liver fluke *Opisthorchis viverrini* being the foremost risk factor in endemic regions like Thailand, which reports the highest global incidence [10,11,12]. CCA pathogenesis is driven by chronic inflammation and cholestasis, which induce genetic and epigenetic alterations through persistent cytokine exposure, activate pro-survival (ERK1/2, Akt, and NF-κB) and pro-angiogenic (TGF-β, VEGF, and HGF) pathways, and promote EMT, metabolic reprogramming (c-Met, GLUT-1, and NIS), and stromal remodeling to enable invasion and metastasis [13]. With only a minority of patients eligible for curative resection, treatment has shifted toward systemic therapies—chemotherapy, FGFR and IDH1 inhibitors, and immunotherapy—and locoregional approaches anchored in precision medicine to overcome CCA’s molecular complexity and therapeutic resistance [14]. Despite advancements in systemic therapies, surgical resection remains the only curative option, albeit feasible in a minority of cases. Therefore, there is an urgent and continuous need for in-depth research to elucidate drug resistance mechanisms and develop novel therapeutic strategies, including precision medicine approaches, to improve outcomes for patients afflicted with CCA.

**Figure 1:**
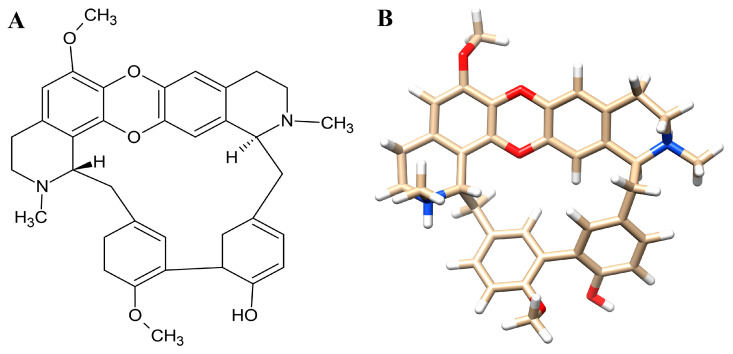
The 2D (**A**) and 3D (**B**) chemical structure of tiliacorinine.

Most new anticancer and anti-infective drugs derive from natural products, especially medicinal plants, underscoring biodiversity and ethnopharmacology’s pivotal role in drug discovery [15]. *Tiliacora triandra* (Colebr.) Diels, known as Ya-Nang, is a flowering climber in the Menispermaceae family, which comprises about 64 genera and 379 species, primarily distributed across tropical areas [16]. In traditional medicine, *T. triandra* has been prized for its antioxidant and anti-inflammatory activities [17]. Traditional healers employ *T. triandra* for both cancer prevention and treatment [18]. Although extracts from various parts of *T. triandra* have demonstrated anticancer activity against colon, lung, hepatoma, and CCA cell lines, the molecular targets underlying these effects remain poorly characterized [19,20,21,22]. *T. triandra*’s phytochemical profile is dominated by bisbenzylisoquinoline alkaloids, 2′-Nortiliacorinine, dinklacorine, tiliacorine, tiliacorinine, cocsoline, O-methylmicrathine, trigilletine, (+)-2′-Norcocsuline, nortiliacorine A, tiliacorinin-2′-N-oxide, tiliageine, yanangcorinin, yanangine, and tilitriandrine—alongside notable flavonoids and phenolic acids such as catechin, quercetin, and gallic acid [17,23]. Tiliacorinine is the major bisbenzylisoquinoline alkaloid of *T. triandra*, featuring two ether-linked benzylisoquinoline units [24]. The chemical structures in both 2D and 3D formats are presented in Figure 1. The crude extract of *T. triandra* and its alkaloid tiliacorinine both exhibited dose-dependent cytotoxicity against A549 lung cancer cells, with IC_50_ values of 48.25 µg/mL and 26.38 µg/mL, respectively [25]. Tiliacorinine induces apoptosis in cholangiocarcinoma cells in vitro and significantly inhibits tumor growth in xenograft mouse models [22]. In 2023, Detarya et al. reported that tiliacorinine induces apoptosis in cholangiocarcinoma cells by downregulating the anti-apoptotic proteins Bcl-xL and XIAP, activating caspase-3, caspase-9, and PARP, and inhibiting phosphorylated Akt and STAT3. Their docking studies and EGF/EGFR activation model identified EGFR, PI3K/Akt, JAK2, and the STAT3 SH2 domain as key molecular targets, and tiliacorinine synergized with gemcitabine to restore its cytotoxicity in resistant cells [26]. However, the molecular mechanisms underlying tiliacorinine’s anticancer effects in CCA remain poorly understood. To fill this gap, we applied an integrative network pharmacology strategy combined with molecular docking and molecular dynamics simulations to elucidate its potential targets and therapeutic pathways. Network pharmacology integrates systems biology, bioinformatics, and network science to map drug–target–disease interactions, enabling identification of key nodes and pathways for drug discovery [27,28]. This approach is particularly valuable in traditional medicine, revealing active compounds and their mechanisms of action [29]. Combined with molecular docking, it allows in silico prediction of ligand–target binding, reducing costs and reliance on animal testing through the 3Rs principle [30,31]. Molecular docking further aids in validating key interactions by evaluating binding energy and active sites [32]. This study employs network pharmacology, molecular docking, and molecular dynamics to investigate the mechanisms of tiliacorinine in cholangiocarcinoma (CCA) treatment.

## 2. Materials and Methods

### 2.1. Assessment of Drug Likeness and ADMET Properties of Tiliacorinine

The drug-likeness of tiliacorinine was evaluated against Lipinski’s Rule of Five—monitoring molecular weight (≤500 Da), lipophilicity (LogP ≤ 5), topological polar surface area (TPSA), hydrogen bond donors (≤5) and acceptors (≤10), rotatable bonds, and predicted aqueous solubility—to ensure properties conducive to passive membrane diffusion. A comprehensive ADMET profile was generated by submitting the compound’s SMILES notation to the SwissADME (http://www.swissadme.ch; accessed on 10 April 2025) and PkCSM platforms (https://biosig.lab.uq.edu.au/pkcsm/prediction; accessed on 10 April 2025). SwissADME predicted drug-likeness profiles, gastrointestinal absorption, P-glycoprotein substrate likelihood, bioavailability score, synthetic accessibility, water solubility, and blood–brain barrier permeation, while PkCSM provided quantitative estimates for Caco-2 permeability, human intestinal absorption, volume of distribution, plasma protein binding, CYP450 enzyme interactions, total clearance, renal OCT2 substrate status, hERG-mediated cardiotoxicity risk, and hepatotoxicity potential. Together, these in silico assessments of oral bioavailability and drug-likeness furnish a robust framework for prioritizing tiliacorinine for subsequent in vitro and in vivo studies [33,34].

### 2.2. Target Proteins of Tiliacorinine

The chemical structure of tiliacorinine was obtained in canonical SMILES format from the PubChem database (https://pubchem.ncbi.nlm.nih.gov/; accessed on 10 April 2025), ensuring precise stereochemical representation and connectivity information. SMILES string was then submitted to the SwissTargetPrediction web server (http://www.swisstargetprediction.ch; accessed on 10 April 2025), which combines 2D similarity and 3D pharmacophore models to forecast the most likely human protein targets of small molecules. The SEA (https://sea.bkslab.org; accessed on 10 April 2025/) and SuperPred (https://prediction.charite.de/; accessed on 10 April 2025) databases were used to predict tiliacorinine’s protein targets. These predictions were then curated by mapping each target to its UniProt accession, standardizing gene names, protein symbols, and species annotations while removing redundant or low-confidence entries. Target duplicates were eliminated to ensure accuracy and prevent redundancy. This harmonized dataset ensured consistent annotation and seamless integration with pathway enrichment and network pharmacology analyses for elucidating tiliacorinine’s mechanism of action [35].

### 2.3. Potential Targets in Cholangiocarcinoma (CCA)

A focused gene set of “cholangiocarcinoma (CCA)” associated candidates was assembled by extracting entries from GeneCards (https://www.genecards.org/; accessed on 10 April 2025) based on relevance score thresholds to prioritize high-confidence tumorigenic drivers. Target genes were restricted to *Homo sapiens*. The predicted protein targets of tiliacorinine were then overlapped with this CCA gene list using the InteractiVenn (https://www.interactivenn.net/; accessed on 10 April 2025) Venn tool, pinpointing the shared nodes that represent potential points of therapeutic intervention [36]. By isolating these intersecting proteins, we generated a refined panel of high-priority targets for further pathway enrichment, network pharmacology analysis, and experimental validation of tiliacorinine’s anticancer effects.

### 2.4. Protein–Protein Interaction Network (PPI)

Protein–protein interactions (PPIs) underlie the regulation of virtually all cellular processes and provide key insights into the architecture of biological networks. To explore how tiliacorinine might modulate proteins implicated in CCA, we constructed a PPI network using STRING (version 12.0; http://string-db.org; accessed on 10 April 2025). Only human proteins were included, and edges with a confidence score > 0.4 were retained to ensure medium confidence in the predicted interactions. The resulting network was imported into Cytoscape (version 3.10.3; https://cytoscape.org; accessed on 10 April 2025) for visualization and analysis. We then applied the cytoHubba plugin (version 0.1; https://apps.cytoscape.org/apps/cytohubba; accessed on 10 April 2025) to rank proteins by degree centrality, thereby identifying the most interconnected “top 10 hub” targets within the network [37].

### 2.5. Gene Ontology (GO) and Kyoto Encyclopedia of Genes and Genomes (KEGG) Enrichment Analysis

Gene Ontology (GO) enrichment was performed using ShinyGO version 0.82 (http://bioinformatics.sdstate.edu/go/; accessed on 5 May 2025) to annotate functional themes among the identified targets. Only *H. sapiens* genes were included, and terms with a false discovery rate (FDR) ≤ 0.05 were considered significant. Analysis was restricted to the top 20 enriched GO terms (maximum background size: 2000 genes) and visualized as plots ranked by fold enrichment. Enriched terms were organized into three GO categories, including molecular function (MF), biological process (BP), and cellular component (CC), to reveal the tiliacorinine compound’s impact on molecular activities, biological pathways, and subcellular localization. Finally, we assessed how tiliacorinine modulates these GO categories to infer its potential functional implications. To explore how tiliacorinine influences CCA signaling networks, we performed KEGG pathway enrichment analysis in ShinyGO. We applied the same FDR cutoff and pathway-size filters used for our GO study to maintain consistency. For each significantly enriched KEGG pathway, we then counted the number of genes meeting these criteria, thereby mapping the molecular circuits through which tiliacorinine may exert therapeutic effects in CCA [38].

### 2.6. Molecular Docking Analysis

Three-dimensional protein structures were retrieved from the RCSB Protein Data Bank (https://www.rcsb.org; accessed on 5 May 2025) [39]. and filtered for resolutions better than 3 Å to ensure high accuracy for CCA studies. Using BIOVIA Discovery Studio 2025, all bound ligands and crystallographic water molecules were removed, and the cleaned coordinates were saved as PDB files. Protonation states and ion charges were then adjusted to physiological pH 7.0 in AutoDock Tools (ADT) version 4.2.6, with the resulting structures exported in both PDB and PDBQT formats. Tiliacorinine compound structures were downloaded from PubChem (http://pubchem.ncbi.nlm.nih.gov/) in SDF format [40], converted to PDB, and hydrogen atoms were added. Atomic charges were assigned via the AM1-BCC algorithm within the AMBER force field, and molecule energy was minimized in UCSF Chimera version 1.10.19 using the steepest descent and conjugate gradient steps for structural refinement [41]. Docking grid definitions centered on each protein’s active site were generated with AutoGrid 4.2. Molecular docking simulations were carried out in AutoDock 4.2.6 using the Lamarckian genetic algorithm (50 GA runs, population size 200) [42]. Details of the selected proteins and ligands, including their box dimensions, are provided in Supplementary Table S2. Positive control compounds were mainly selected from co-crystallized ligands of the target proteins in the PDB, ensuring experimentally validated binding modes for benchmarking our docking protocol. When no co-crystallized ligand was available, we used alternative ligands or inhibitors with reported interactions. Thus, all reference compounds had either crystallographic evidence or strong literature support for their relevance to the studied targets. As described in Supplementary Table S2, we re-docked the co-crystallized ligands into their respective protein binding sites to confirm the reliability of the grid parameters and the accuracy of the scoring functions. Final poses were ranked by binding energy and inspected for key interactions. Detailed binding modes and chemical bond networks were then analyzed in AutoDock 4 and BIOVIA Discovery Studio 2025 [43].

### 2.7. Molecular Dynamics Simulation Studies

Molecular dynamics (MD) simulations were used to capture the time-dependent behavior of the MTOR-tiliacorinine and MTOR-GDC-0980 complexes, illuminating how these ligands influence protein flexibility and binding. Each complex was first prepared at pH 7.0 using a protein-preparation wizard: hydrogens were added, bond orders assigned, missing side chains and loops rebuilt, hydrogen-bond networks optimized, and water orientations sampled. Systems were then solvated in an orthorhombic box (10 Å × 10 Å × 10 Å) of TIP3P water, neutralized with Na^+^ and Cl^−^ ions to 0.15 M, and parameterized for simulation. Production runs were carried out for 300 ns under an NPT ensemble at 310 K and pressure at 1.01 bar. Long-range electrostatics employed the Smooth Particle Mesh Ewald method (PME), while solvent was modeled with a simple point-charge representation. Trajectory analyses performed via the Simulation Interaction Diagram wizard included ligand–protein contact maps and root-mean-square deviation (RMSD) profile, Root Mean Square Fluctuation (RMSF) plots and MD timeline interaction analysis for both ligand and protein atoms. All MD simulations and subsequent analyses were conducted using Desmond from Schrödinger. These outputs provided a detailed picture of structural stability, conformational shifts, and key interaction hotspots over the full simulation period [44].

## 3. Results

### 3.1. Prediction of Drug-Likeness and ADMET Characteristics of Tiliacorinine

Drug-likeness assessment filters candidate therapeutics by evaluating how their physicochemical and pharmacokinetic properties align with established oral-bioavailability guidelines. As summarized in Table 1, tiliacorinine (C_36_H_36_N_2_O_5_) has a molecular weight of 576.68 g/mol, two rotatable bonds, and 24 aromatic heavy atoms, which satisfy Veber’s rule on flexibility and Egan’s aromaticity criteria. It carries seven hydrogen bond acceptors and one donor, both within Lipinski’s thresholds of ≤10 and ≤5, respectively, while its topological polar surface area (63.63 Å^2^) lies well below the 140 Å^2^ cut-off, suggesting good membrane permeability. A consensus Log P of 5.24 denotes moderate lipophilicity, which may enhance membrane partitioning but underlies the compound’s poor aqueous solubility. Despite slightly exceeding Lipinski’s molecular-weight recommendation, tiliacorinine meets the core requirements of Lipinski’s rule-of-five (aside from MW), Veber’s rule, and the Egan filter, indicating promising oral bioavailability potential, provided formulation strategies can address its hydrophobicity.

Pharmacokinetic predictions from pKCSM (Table 2) indicate that tiliacorinine, despite low aqueous solubility and limited skin permeability (log Kp = −2.735), achieves moderate Caco-2 permeability (log Papp = 0.63) and high human intestinal absorption (93.6%), supporting efficient oral uptake. It functions as both a P-glycoprotein substrate and inhibitor (types I and II), suggesting active efflux and possible drug–drug interactions.

In terms of distribution and elimination, tiliacorinine shows limited tissue penetration, no predicted blood–brain barrier or CNS permeability, moderate clearance, and no involvement with the renal OCT2 transporter. Metabolically, it is a CYP3A4 substrate without inhibiting major CYP450 isoforms, pointing to minimal metabolic interaction risk. Toxicity flags include potential mutagenicity (positive Ames test), hERG channel inhibition (cardiotoxic risk), and moderate acute toxicity (LD_50_). No hepatotoxicity or skin sensitization is predicted, though environmental toxicity toward *T. pyriformis* and minnows is noted. These in silico findings should be confirmed by experimental studies.

### 3.2. Target Proteins Identification and Analysis

**Figure 2:**
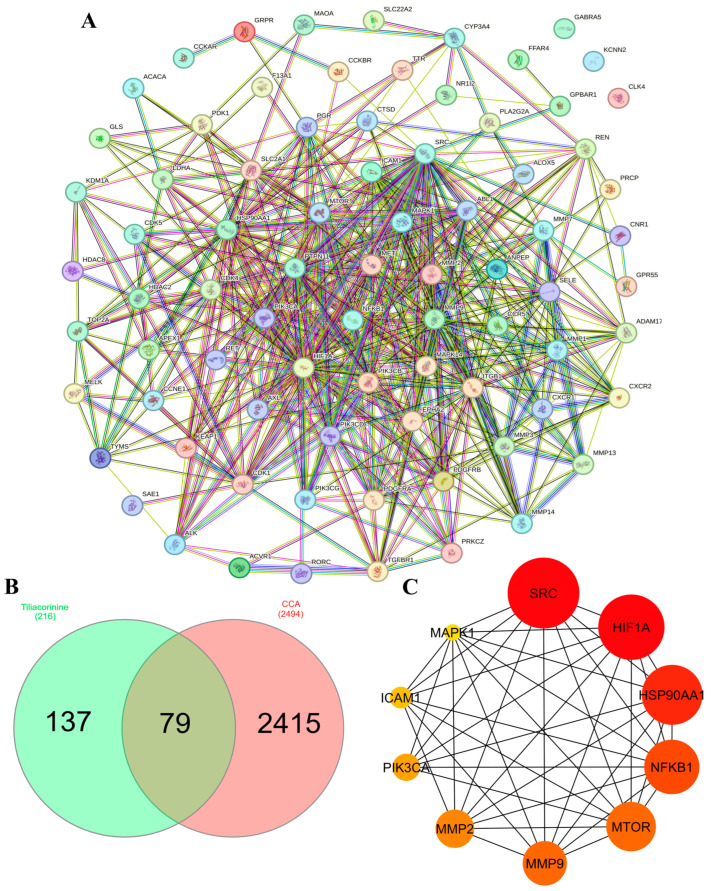
Network pharmacology analysis of Tiliacorinine and CCA against colon cancer. (**A**) Protein–protein interaction network of these shared targets, constructed using the STRING database. (**B**) Venn diagram illustrates the overlap of predicted targets for Tiliacorinine and CCA. (**C**) Top 10 hub proteins in the *H. sapiens* PPI network, ranked by degree centrality using the CytoHubba plugin in Cytoscape.

We first used SwissTargetPrediction, SEA, and SuperPred database to predict 212 molecular targets for tiliacorinine. Meanwhile, 2494 cholangiocarcinoma (CCA) associated genes were retrieved from the GeneCards database. After removing duplicates, intersection analysis revealed 79 common targets (Figure 2B), representing potential therapeutic nodes through which tiliacorinine may modulate key drivers of CCA pathogenesis. These 79 overlapping targets were then designated as tiliacorinine’s CCA targets for further investigation.

### 3.3. PPI Network Construction and Hub Gene Identification

To explore how the predicted targets interact, we built a protein–protein interaction network in the STRING database, incorporating all 79 candidate proteins. Figure 2A illustrates this network, where each node denotes a protein, edges represent interactions, and line thickness corresponds to interaction strength. The CytoHubba plugin in Cytoscape 3.10.3 was then applied to rank proteins by their degree of connectivity and highlight the top ten hub genes. In Figure 2C, node color intensity reflects connectivity scores (darker red indicates higher centrality). The top ten hub genes were SRC Proto-Oncogene (SRC), Hypoxia Inducible Factor 1 Subunit Alpha (HIF1A), Heat Shock Protein 90 Alpha Family Class A Member 1 (HSP90AA1), Nuclear Factor Kappa B Subunit 1 (NFKB1), Mechanistic Target Of Rapamycin Kinase (MTOR), Matrix Metallopeptidase 9 (MMP9), Matrix Metallopeptidase 2 (MMP2), Phosphatidylinositol-4,5-Bisphosphate 3-Kinase Catalytic Subunit Alpha (PIK3CA), Intercellular Adhesion Molecule 1 (ICAM1), and Mitogen-Activated Protein Kinase 1 (MAPK1). These central nodes likely drive key signaling pathways and are strong candidates for mediating the anti-cholangiocarcinoma activity of tiliacorinine, warranting further experimental validation.

### 3.4. GO and KEGG Enrichment Analyses

**Figure 3:**
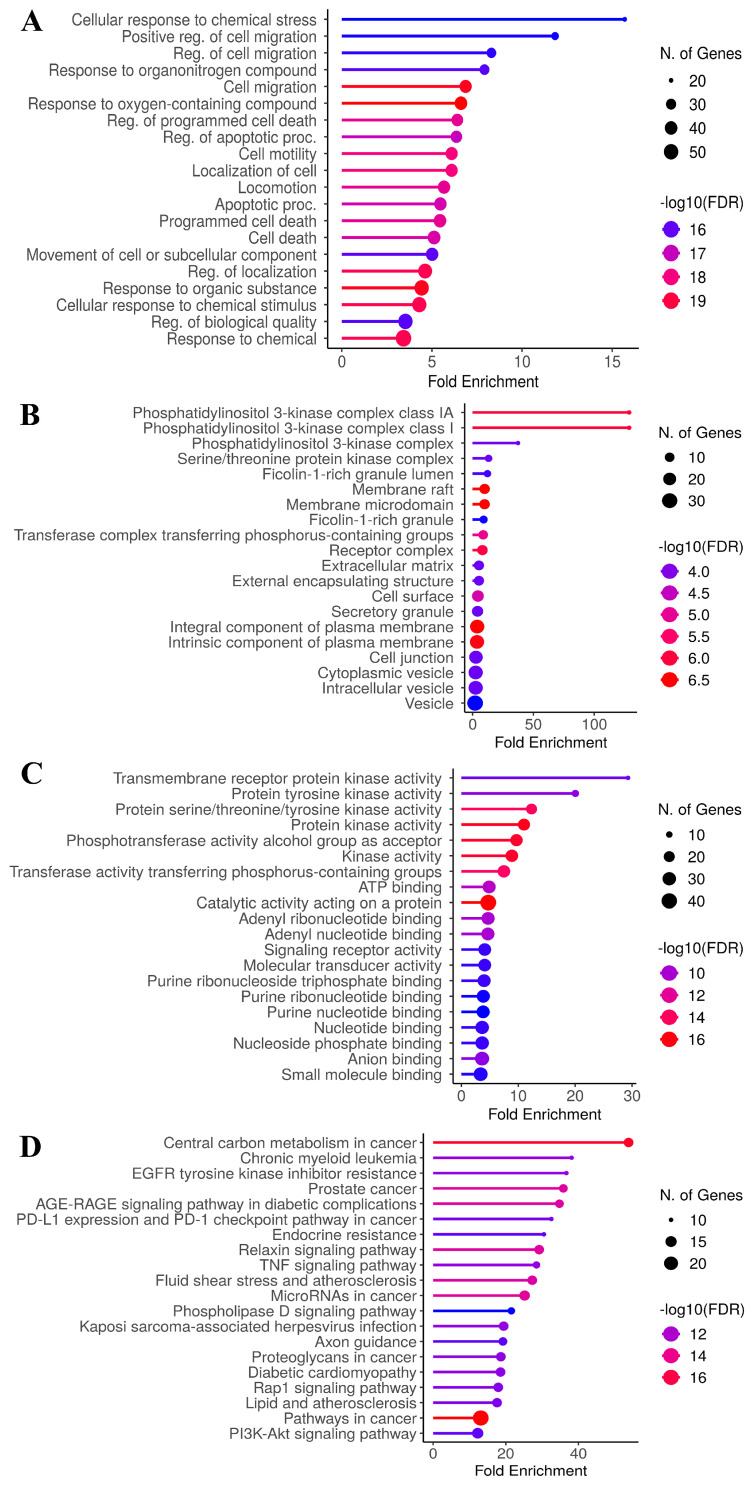
Gene Ontology (GO) and Kyoto Encyclopedia of Genes and Genomes (KEGG) enrichment analysis of 79 identified candidate targets. This figure illustrates the enrichment analysis results for targets influenced by bioactive compounds Tiliacorinine and CCA treatment. The analysis was conducted with a *p* value < 0.05. Specifically, the figure displays (**A**) GO Biological process enrichment, (**B**) GO Cellular component enrichment, (**C**) GO Molecular function enrichment, and (**D**) KEGG pathway analysis.

Optimized enriched proteins were imported into the database for annotation, visualization, and integrated discovery for KEGG and GO analyses, with relevant pathways visualized based on the number of enriched proteins (*p* < 0.05). Gene Ontology (GO) enrichment analysis was performed across three categories: biological processes (BP), cellular components (CC), and molecular functions (MF). The top 20 enriched terms were identified for each category, with emphasis placed on their relevance to CCA. In the biological process category (Figure 3A), the five most significantly enriched terms included response to oxygen-containing compound (GO:1901700), response to organic substance (GO:0010033), cell migration (GO:0016477), regulation of localization (GO:0032879), and response to chemical (GO:0042221). For the cellular component category (Figure 3B), the top five enriched terms were integral components of plasma membrane (GO:0005887), membrane raft (GO:0045121), membrane microdomain (GO:0098857), intrinsic component of plasma membrane (GO:0031226) and phosphatidylinositol 3-kinase complex class IA (GO:0005943). In the molecular function category (Figure 3C), the top five terms included catalytic activity acting on a protein (GO:0140096), protein kinase activity (GO:0004672), phosphotransferase activity for an alcohol group as an acceptor (GO:0016773), kinase activity (GO:0016301) and protein serine/threonine/tyrosine kinase activity (GO:0004712).

To elucidate the signaling mechanisms underlying the effects of tiliacorinine on cholangiocarcinoma (CCA), KEGG pathway enrichment analysis was performed on the overlapping molecular targets. The five most significantly enriched pathways (Figure 3D) included: pathways in cancer (hsa05200), central carbon metabolism in cancer (hsa05230), microRNAs in cancer (hsa05206), the relaxin signaling pathway (hsa04926), and prostate cancer (hsa05215). These results indicate that tiliacorinine may exert its anti-CCA activity through multiple biological processes and signaling cascades, highlighting its potential as a multi-target therapeutic candidate.

### 3.5. Ligand–Protein Docking Analysis

To assess the reliability of drug-target interactions, molecular docking analysis was conducted on ten hub proteins identified as key therapeutic targets, including SRC, HIF1A, HSP90AA1, NFKB1, MTOR, MMP9, MMP2, PIK3CA, ICAM1, and MAPK1.

**Figure 4:**
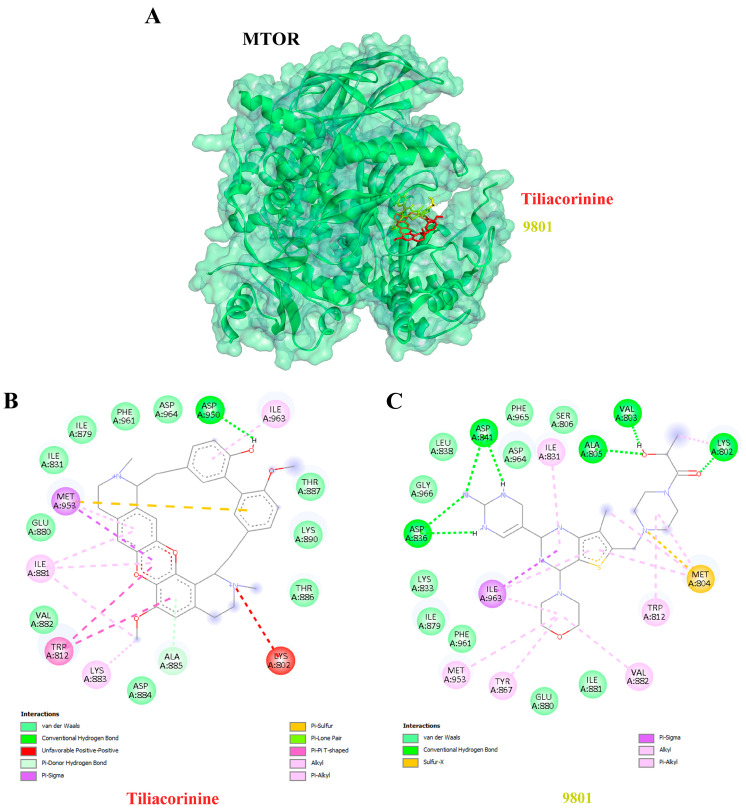
Protein–ligand docking of Tiliacorinine and GDC-0980 with mTOR. (**A**) Three-dimensional view of mTOR bound to Tiliacorinine (red) and GDC-0980 (yellow). (**B**) Two-dimensional interaction map of the Tiliacorinine–mTOR complex. (**C**) Two-dimensional interaction map of the GDC-0980–mTOR complex (positive control).

Affinity describes a ligand’s ability to bind to its target receptor, with more negative affinity values reflecting stronger binding interactions. Among the targets, MTOR exhibited the highest binding affinity for tiliacorinine, with a docking score of −10.78 kcal/mol and an inhibition constant of 12.62 nM (Table 3). Notably, tiliacorinine is bound to the same active site as the established MTOR inhibitor 9801 (Figure 4A) and demonstrated superior binding energy and inhibition constant. In addition, interaction analysis revealed that tiliacorinine engages in a critical hydrogen bond with MTOR at ASP950 and establishes hydrophobic interactions with MET953, TRP812, ILE881, ILE963, ALA885, and LYS883 (Figure 4B), indicating strong target specificity and structural stability.

**Figure 5:**
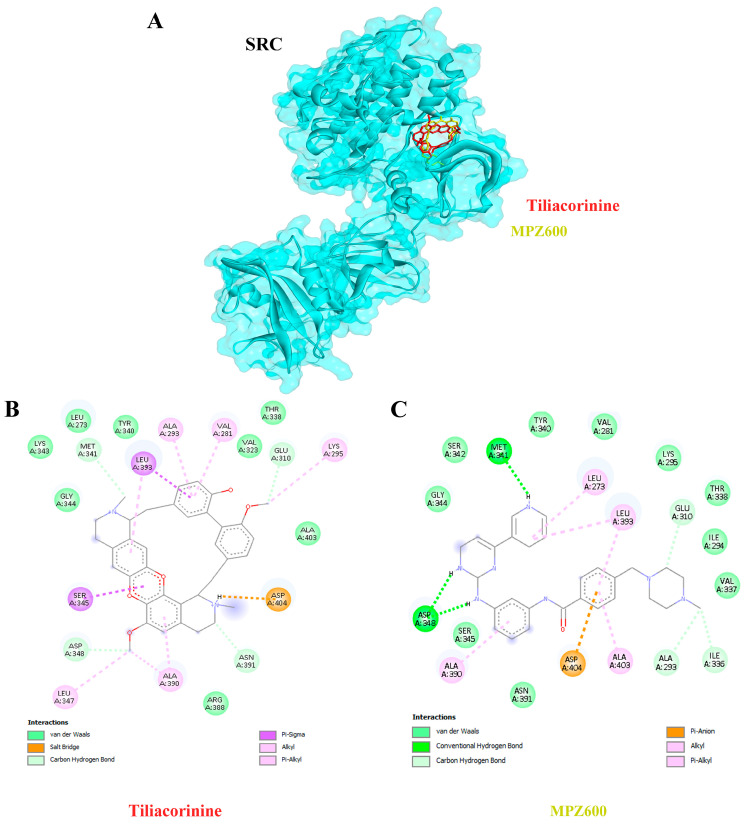
Protein–ligand docking interactions of Tiliacorinine and MPZ600 with SRC. (**A**) Three-dimensional view of SRC protein docked with Tiliacorinine (red) and MPZ600 (yellow). (**B**) Two-dimensional interaction map for Tiliacorinine–SRC binding. (**C**) Two-dimensional interaction map for MPZ600–SRC binding (positive control).

**Figure 6:**
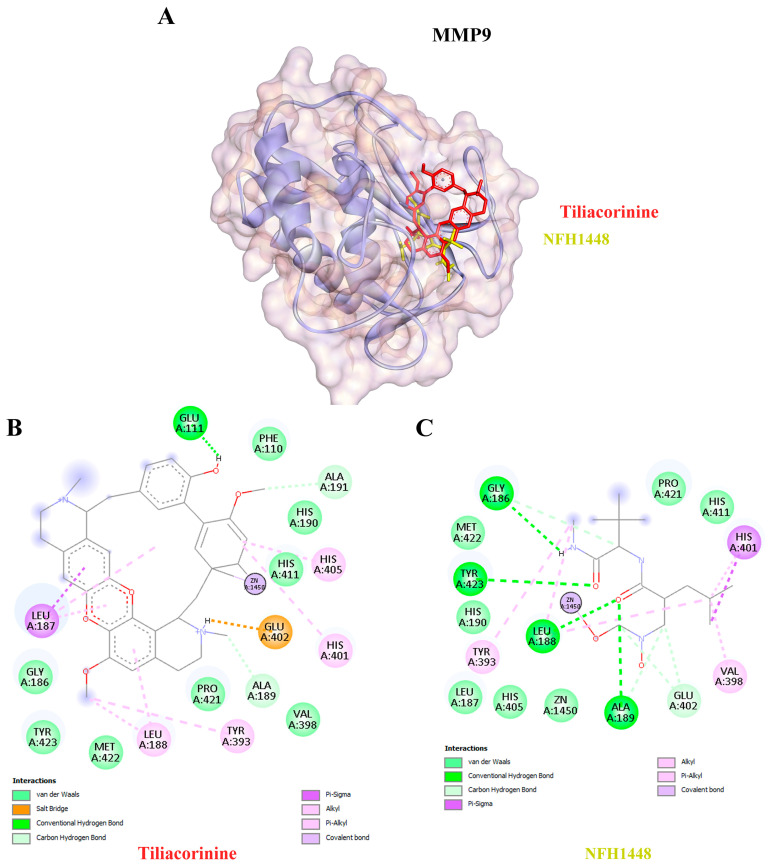
Visualization of MMP9 protein-Ligand Docking Interactions. (**A**) Three-dimensional representation of Tiliacorinine (highlighted in red) and NFH1448 (highlighted in yellow) docked with MMP9. (**B**) Two-dimensional illustration of the docking interaction between Tiliacorinine and MMP9. (**C**) Two-dimensional depiction of the docking interaction of NFH1448 (used as a positive control) with MMP9.

**Figure 7:**
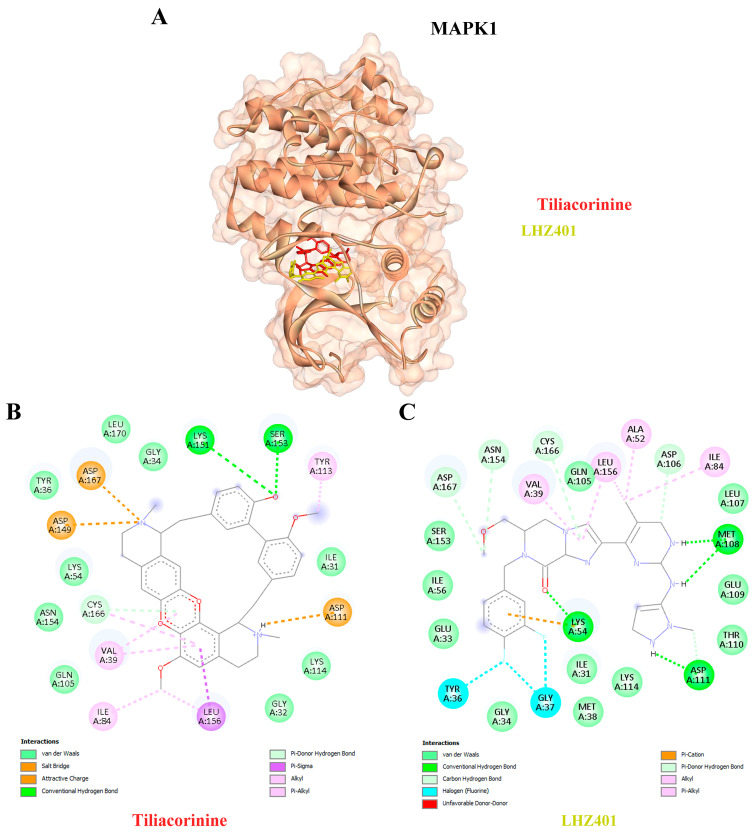
Visualization of MAPK1 protein-Tiliacorinine Docking Interactions. (**A**) Three-dimensional representation of Tiliacorinine (highlighted in red) and LHZ401 (highlighted in yellow) docked with MAPK1. (**B**) Two-dimensional illustration of the docking interaction between Tiliacorinine and MAPK1. (**C**) Two-dimensional depiction of the docking interaction of LHZ401 (used as a positive control) with MAPK1.

Moreover, tiliacorinine exhibited strong binding affinities with key protein targets SRC, MMP9, and MAPK1, achieving docking scores of −9.29, −9.42 and −9.30 kcal/mol, respectively (Table 3). In comparison, the reference inhibitors MPZ600, NFH1448 and LHZ401 demonstrated lower binding affinities, with docking scores of −8.77, −7.95 and −8.23 kcal/mol, respectively. As shown in Figure 5A,B, tiliacorinine binds within the active site of SRC, overlapping with the binding region of the reference inhibitor MPZ600. It forms four hydrogen bonds with key amino acid residues, contributing to its binding stability. Furthermore, tiliacorinine demonstrated three hydrogen bond interactions with GLU111, ALA189, and ALA191 in the same binding pocket as the known MMP9 inhibitor NFH1448 (Figure 6B). As shown in Figure 7A, tiliacorinine binds to MAPK1 at the same pocket as the known inhibitor LHZ401, forming two hydrogen bonds with residues LYS151 and SER153 (Figure 7B). Collectively, these findings highlight tiliacorinine’s strong binding affinities to pivotal cholangiocarcinoma-related targets, including MTOR, SRC, MMP9, and MAPK1, underscoring its potential as a promising multi-target therapeutic candidate.

### 3.6. Analysis of Molecular Dynamics Trajectories 

**Figure 8:**
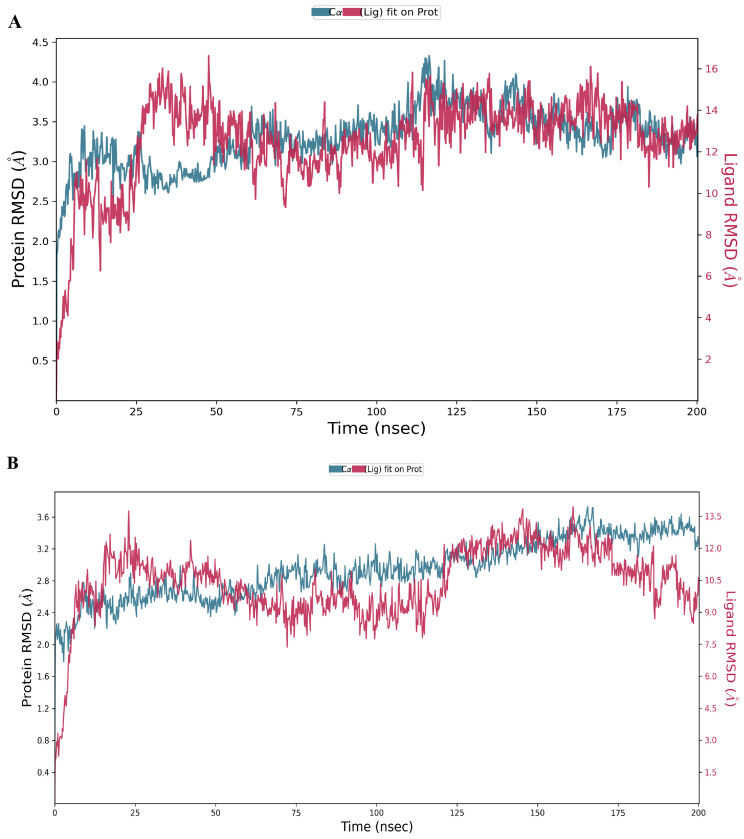
Molecular Dynamics Simulation Analysis of Tiliacorinine and GDC-0980 with MTOR over a 200 ns trajectory. (**A**,**B**) Root Mean Square Deviation (RMSD) profiles illustrating the protein–ligand interaction stability during MD simulations of Tiliacorinine with MTOR (**A**) and GDC-0980 with MTOR (**B**).

Tiliacorinine was identified as the most promising candidate based on its strong interactions with key MTOR binding residues and high docking score. Its interaction profile was further examined via 200 ns molecular dynamics simulations, alongside the known MTOR inhibitor GDC-0980. RMSD and RMSF are key indicators of the structural stability of docked ligand–protein complexes. Higher RMSD values reflect greater deviations from the initial structure, indicating reduced complex stability. The MTOR–tiliacorinine complex showed initial RMSD fluctuations between 0.5 and 4.0 Å during the first 30 ns, followed by stable behavior ranging from 2.5 to 4.0 Å through 200 ns (Figure 8A). This indicates a well-equilibrated and structurally stable complex after the initial relaxation phase. Similarly, the MTOR–GDC-0980 complex displayed RMSD shifts of 0.8–3.6 Å up to 25 ns before stabilizing. Then, ligand–protein complexes demonstrated overall structural stability, with early fluctuations between 2.0 and 3.6 Å (Figure 8B).

RMSF measures local flexibility along the protein chain. Regions with persistent α-helices and β-strands (≥70% of the simulation) are highlighted in red and blue, respectively. The tiliacorinine ligand–MTOR protein complex displayed RMSF values (within 0.8–3.2 Å) comparable to the GDC-0980-MTOR structure (0.8–4.0 Å), indicating similar flexibility (Supplement Figure S1A,B). Most residue fluctuations remained below 3.0 Å, reflecting limited atomic displacement and supporting the structural stability of both complexes. Elevated RMSF peaks, primarily in loop regions, indicated moderate flexibility, while the α-helical and β-strand segments (marked in red and blue) retained consistent secondary structures throughout the simulation.

The MD simulations demonstrated stable profiles for both complexes, with consistent radius of gyration (rGyr) values indicating preserved global compactness of MTOR, while molecular surface area (MolSA) and solvent-accessible surface area (SASA) showed only minor fluctuations, reflecting steady solvent exposure of the ligands and surrounding residues. Polar surface area (PSA) values also remained stable, supporting persistent polar interactions at the ligand–MTOR interface (Supplement Figure S2A,B). Collectively, these descriptors, together with the RMSD results, confirm that both tiliacorinine and GDC-0980 maintained stable binding within the MTOR active site, with limited structural drift and sustained residue flexibility, thereby reinforcing the robustness of the docking and MD simulations.

**Figure 9:**
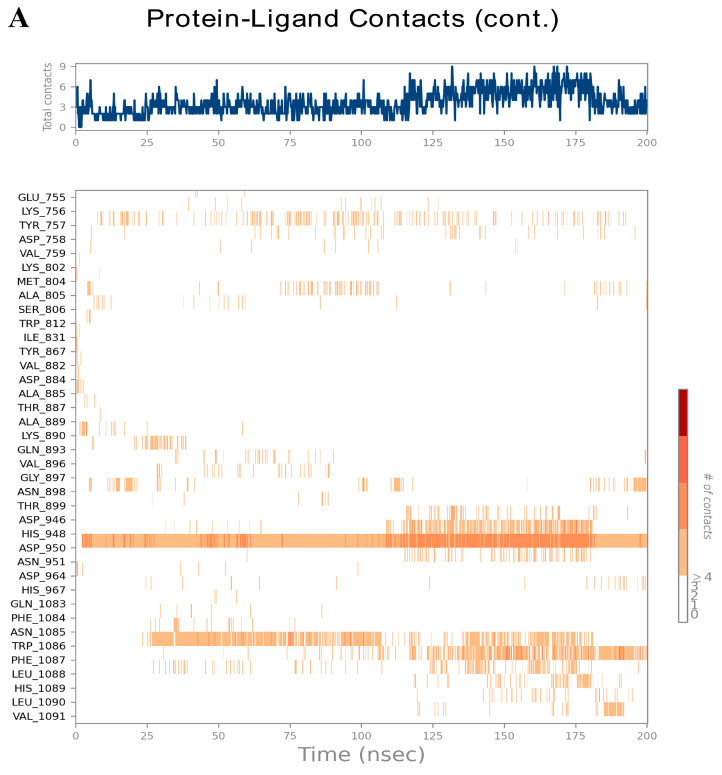

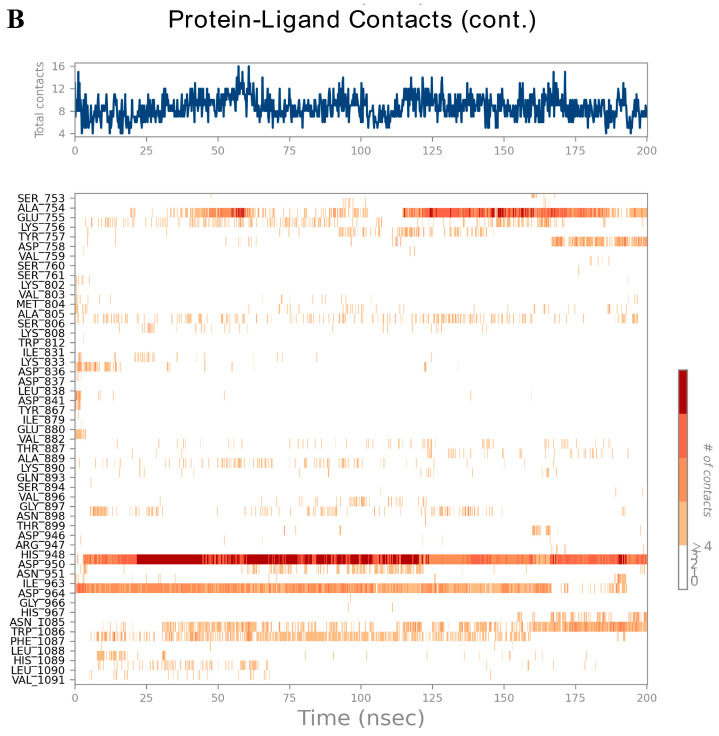
MD timeline interaction analysis of Tiliacorinine and GDC-0980 with MTOR over a 200 ns simulation. Post-simulation assessments illustrate the total contact timelines and amino acid interactions of Tiliacorinine (**A**) and GDC-0980 (**B**) with MTOR.

**Figure 10:**
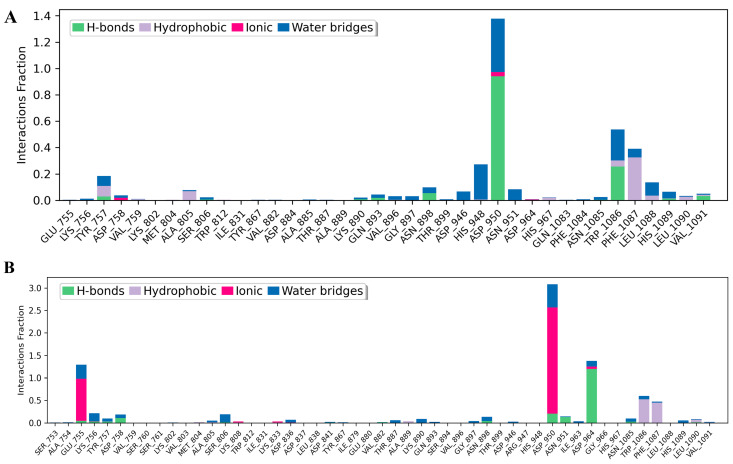
Molecular dynamics (MD) timeline interaction analysis of tiliacorinine and GDC-0980 with MTOR over a 200 ns simulation, accompanied by protein–ligand contact plots. Panels (**A**,**B**) show the interacting residues and their interaction fractions throughout the trajectory for the tiliacorinine–MTOR and GDC-0980–MTOR complexes, respectively.

The contact timelines in Figure 9A,B illustrate residue–ligand interactions across the 200 ns simulation. Darker orange shades indicate residues forming multiple simultaneous contacts. Tiliacorinine engaged MTOR residues ASP950, TRP1086, and PHE1087 (Figure 9A), while the MTOR–GDC-0980 complex showed stable interactions at ASP950, ASP964, TRP1086, and PHE1087 (Figure 9B), reflecting consistent binding throughout the trajectory. Histograms in Figure 10A,B depict intermolecular interactions at MTOR’s active site, including hydrogen bonding, hydrophobic contacts, ionic interactions, and water bridges. In the MTOR-tiliacorinine complex (Figure 10A), hydrogen bonds were formed at ASP950 and TRP1086, while PHE1087 and ASP948 participated in hydrophobic and water-mediated interactions. The MTOR-GDC-0980 complex (Figure 10B) showed key contacts at ASP964, ASP950, and ASN951, with ionic interactions involving GLU755 and ASP950. These MD simulation results align well with docking predictions, reinforcing tiliacorinine’s stable and specific binding at the MTOR active site.

## 4. Discussion

Cancer remains a leading global health challenge, with nearly 20 million new cases and 9.7 million deaths reported in 2022 [1,45]. Among these, CCA incidence is rising, particularly in Western countries, with male mortality rates increasing in Europe over recent decades [46]. Southeast Asia, especially Thailand, bears a disproportionately high burden of CCA [7]. Conventional CCA cancer therapies are hindered by toxicity, low selectivity, and resistance issues [47]. Thai traditional medicine, with its diverse bioactive compounds, offers a promising alternative approach for more effective and targeted treatment. Tiliacorinine, an alkaloid from *T. triandra*, has gained attention for its anticancer potential, particularly against CCA [26]. Despite slightly exceeding Lipinski’s recommended molecular weight, it retains favorable oral drug-like properties—low TPSA, limited rotatable bonds, and adequate hydrogen bonding—predicting good membrane permeability and high intestinal absorption. However, poor aqueous solubility and dual P-glycoprotein substrate/inhibitor activity raise concerns about tumor uptake and drug–drug interactions. To address the issue of poor aqueous solubility, several formulation strategies may be considered, including nanoparticle-based delivery systems, salt formation, and co-crystallization techniques, all of which have the potential to enhance solubility and bioavailability. Lipinski’s Rule of Five provides key guidelines for evaluating the drug-likeness of compounds, especially for oral administration. It states that orally active drugs typically meet at least three of the following criteria: ≤5 hydrogen bond donors, ≤10 hydrogen bond acceptors, molecular weight < 500 daltons, and ClogP ≤ 5 [48]. These physicochemical traits help predict a compound’s absorption and membrane permeability, which are critical for oral bioavailability [49]. ADMET predictions indicate tiliacorinine is a CYP3A4 substrate with minimal CYP inhibition, low CNS penetration (appropriate for CCA treatment), and safety flags including AMES positivity and potential hERG inhibition-necessitating further experimental validation. The positive AMES prediction for tiliacorinine indicates potential to cause DNA mutations, raising concerns about mutagenic or carcinogenic liability, while the predicted hERG II inhibition suggests possible interference with the hERG potassium channel, essential for normal cardiac function. Such inhibition can prolong the QT interval, a recognized risk factor for ventricular arrhythmias and associated cardiotoxicity. These safety-related endpoints should be regarded as preliminary alerts, as in silico predictions, though valuable for early screening, have inherent limitations. Therefore, further validation using complementary computational platforms indicated potential safety concerns. ProTox 3.0 predicted mutagenic activity with a probability of 0.77 (Supplement Table S1). Moreover, admetSAR results showed AMES toxicity with a probability of 0.8664, classified the compound as non-carcinogenic with a probability of 0.9451, and identified it as a weak hERG inhibitor with a probability of 0.7513 (Supplement Table S1). However, experimental assays such as hERG patch-clamp and AMES tests are necessary to confirm the translational relevance of these findings. Additionally, structural modifications—such as the incorporation of polar functional groups or the use of bioisosteric replacements—could help mitigate mutagenic risks and reduce the likelihood of hERG channel inhibition. ADMET prediction, alongside Lipinski’s Rule of Five, is essential in early drug discovery to evaluate a compound’s pharmacokinetic profile and clinical viability. Accurate forecasting helps identify candidates with optimal systemic exposure, reducing development risks and improving safety and efficacy [50]. As innovation demands rise and R&D costs grow, ADMET modeling has become a standard tool for prioritizing promising drugs [51]. Together with Lipinski’s Rule of Five and ADMET modeling, these findings support tiliacorinine’s therapeutic promise while highlighting formulation and safety challenges to address in early-phase development.

In this study, the active compound tiliacorinine and its potential targets related to CCA were identified using SwissTargetPrediction, SEA, and SuperPred databases. A total of 2494 CCA-associated genes were collected from the GeneCards database, and intersection analysis revealed 79 overlapping targets (Figure 2B). The GO functional enrichment study revealed that the hub genes were significantly associated (*p* < 0.05) with biological processes and molecular functions including cell migration, integral components of the plasma membrane, catalytic activity on proteins, protein kinase activity, and serine/threonine/tyrosine kinase activity. The KEGG pathways related to the hub genes further indicated their involvement in pathways related to cancer, central carbon metabolism in cancer, and microRNA regulation in cancer. The insights derived from these pathways suggest promising therapeutic targets and strategies to suppress cancer proliferation, metastasis, and drug resistance, while improving treatment efficacy. Nevertheless, the core molecular mechanisms underlying the inhibitory effects of tiliacorinine on CCA remain inadequately characterized. To address this gap, we designed computational experiments to identify key target proteins and relevant pathways modulated by tiliacorinine in CCA.

Protein–protein interaction (PPI) network analysis of the overlapping targets between tiliacorinine and cholangiocarcinoma identified SRC, HIF1A, HSP90AA1, NFKB1, MTOR, MMP9, MMP2, PIK3CA, ICAM1, and MAPK1 as central hub genes (Figure 2C). These nodes exhibited extensive connectivity with surrounding targets, suggesting a high degree of network centrality and functional relevance in key molecular pathways. SRC is a non-receptor tyrosine kinase that plays a central role in oncogenic signaling of CCA, including cell proliferation, survival, migration, and invasion. Its overactivation has been observed in CCA tissues and cell lines [52,53]. SRC plays a multifaceted oncogenic role in CCA by activating key signaling pathways, interacting with FAK in response to HGF to promote proliferation and invasion, forming a metastatic SRC–Hic-5–AKT cascade, and phosphorylating YAP to drive tumor growth and therapy resistance [53]. Targeting SRC with inhibitors such as dasatinib and NXP900 has demonstrated potent antitumor effects, and when combined with standard chemotherapy, these agents amplify cancer cell death and suppress tumor progression [54]. HIF1A plays a critical role in CCA, with elevated expression levels observed in tumor tissues relative to normal bile ducts. Its upregulation is linked to poorer clinical outcomes, including reduced overall and disease-free survival [55]. Functionally, HIF1A drives cancer progression by mediating hypoxia-induced autophagy, epithelial–mesenchymal transition (EMT), and metastasis, positioning it as an oncogenic driver in CCA [56].

HSP90AA1 plays a key role in stabilizing oncogenic proteins and promoting cancer progression. In CCA, its expression is notably high, detected in 44.6% of intrahepatic and 32.8% of extrahepatic cases, and is strongly linked to poor 5-year survival outcomes [57]. Targeting HSP90 with inhibitors like NVP-AUY922 has demonstrated significant anti-proliferative effects in both cell lines and animal models, highlighting its potential as a therapeutic target [57]. Moreover, pan-cancer analyses reveal that HSP90AA1 contributes to pathways such as immune modulation and angiogenesis, further underscoring its relevance in cholangiocarcinoma biology [58,59]. NFKB1 is abnormally overexpressed in CCA tissues, particularly in cases associated with liver fluke infection, while normal bile ducts show minimal expression [60]. Its activation promotes tumor initiation, progression, and metastasis, and genetic variations in its promoter region may increase cancer risk [61]. Targeting NF-κB with inhibitors like DHMEQ has shown promising anti-tumor effects in both cell lines and animal models, highlighting its potential as a therapeutic target in CCA [60]. The PI3K/AKT/mTOR signaling pathway is frequently activated in cholangiocarcinoma, driving tumor development, progression, and resistance to chemotherapy [62,63]. mTOR inhibitors, including sirolimus and everolimus, have demonstrated anti-tumor effects both in vitro and in vivo, with newer agents targeting mTORC1 and mTORC2 showing improved efficacy [63]. A notable case report highlighted a dramatic response to mTOR-targeted therapy in a patient with intrahepatic cholangiocarcinoma harboring TSC1 and ARID1A mutations, indicating potential oncogenic dependence on mTOR in certain subtypes [62]. MMP2 and MMP9 are key enzymes involved in extracellular matrix degradation, promoting tumor invasion, metastasis, and angiogenesis in cholangiocarcinoma [64]. Their elevated expression in tumor tissues and serum is linked to poor clinical outcomes, including lymph node metastasis and vascular invasion [64]. These metalloproteinases are upregulated by platelet-derived growth factor (PDGF) through the p38/MAPK pathway, which also triggers epithelial–mesenchymal transition [64]. Moreover, dual inhibition of mTORC1/2 suppresses their expression and reduces cell migration, underscoring their role in mTOR-driven metastatic signaling [65]. Collectively, MMP2 and MMP9 serve as both prognostic biomarkers and potential therapeutic targets in cholangiocarcinoma. PIK3CA encodes the p110α subunit of PI3K, a key player in the PI3K/AKT/mTOR pathway, which is often dysregulated in cholangiocarcinoma. A gain-of-function mutation in PIK3CA was linked to acquired resistance to FGFR2 inhibitors in a patient with intrahepatic cholangiocarcinoma, underscoring its role in disease progression and therapy resistance [66]. While more frequently seen in gallbladder carcinoma, PIK3CA mutations also occur in subsets of biliary tract cancers, indicating shared oncogenic pathways across these malignancies [67]. ICAM1 (Intercellular Adhesion Molecule 1) is indeed associated with CCA. Additionally, serum ICAM1 concentrations are significantly higher in CCA patients than in healthy individuals, highlighting its potential as a diagnostic and prognostic biomarker [68]. Promisingly, ICAM1 has also emerged as a therapeutic target, with antibody-drug conjugates demonstrating strong anti-tumor efficacy in preclinical models [69]. MAPK1 plays a critical role in intrahepatic cholangiocarcinoma (ICC) by being recruited by osteopontin (OPN) to phosphorylate β-Catenin at SER675, thereby enhancing nuclear accumulation and activating the Wnt/β-Catenin pathway, key drivers of tumor growth and metastasis [70]. Its dysregulation within the MAPK signaling cascade contributes to inflammation, stress responses, epithelial–mesenchymal transition (EMT), and tumorigenesis in CCA [71]. As a core component of the MAPK/ERK pathway, which is frequently activated in liver and biliary tract cancers, MAPK1 represents a promising therapeutic target [71]. The top 10 targets identified through the network pharmacology approach are significantly implicated in CCA carcinogenesis, progression, tumor growth, angiogenesis, and metastasis, highlighting their potential as promising therapeutic candidates.

Tiliacorinine demonstrated strong binding affinities toward SRC, MMP9, and MAPK1, exhibiting more favorable binding energies than the respective positive controls MPZ600, NFH1448, and LHZ401 (Table 3). In CCA, chronic inflammation and persistent oxidative stress synergistically promote tumor initiation. Injured cholangiocytes and inflammatory cells release mediators that alter cell behavior, while reactive oxygen and nitrogen species (RONS) cause DNA damage and sustain inflammation. This vicious cycle creates a tumor-promoting microenvironment, especially in livers affected by chronic infection or inflammation [72]. In CCA, SRC activation has been linked to enhanced metastatic potential, partly through its interaction with growth factor receptors and downstream MAPK and PI3K/AKT pathways, which are sensitive to oxidative stress. Oxidative stress, driven by elevated reactive oxygen species (ROS), can activate SRC and other redox-sensitive kinases, promoting EMT and chemoresistance in CCA cells [73,74]. Studies also suggest that SRC may mediate ROS-induced signaling cascades, amplifying inflammation and tumor aggressiveness in the biliary tract [73]. MMP9 is frequently overexpressed in CCA tissues and serum and is linked to aggressive tumor behavior and metastasis. In CCA, chronic inflammation and oxidative damage promote a tumor microenvironment where MMP9 is activated, enhancing epithelial–mesenchymal transition (EMT) and immune evasion [73,74]. Studies have shown that oxidative stress-related molecular signatures in CCA include MMP9 among key players in redox-dependent pathways [74]. Oxidative stress activates MAPK signaling, including MAPK1 (ERK), JNK, and p38 kinases, through reactive oxygen species (ROS) production. This activation can lead to both pro-survival and pro-apoptotic effects, depending on the cellular context, and contributes to inflammation, EMT, and tumorigenesis in CCA [71]. These mechanisms align with previous findings on tiliacorinine, which induces apoptosis in CCA cells in vitro and significantly suppresses tumor growth in xenograft mouse models [22]. Tiliacorinine promotes apoptosis by downregulating anti-apoptotic proteins Bcl-xL and XIAP, activating caspase-3, caspase-9, and PARP, and inhibiting phosphorylated Akt and STAT3. Molecular docking and EGF/EGFR pathway modeling identified EGFR, PI3K/Akt, JAK2, and the STAT3 SH2 domain as key molecular targets. Notably, tiliacorinine synergized with gemcitabine to restore its cytotoxic efficacy in resistant CCA cells [26]. These findings suggest that targeting SRC, MMP9, and MAPK1 with agents such as tiliacorinine may offer potent antitumor effects. However, further in vitro and in vivo studies are warranted to validate these therapeutic potentials.

Molecular docking analysis revealed that tiliacorinine exhibits strong binding affinity to mTOR, with the highest docking score (Table 3). Notably, it binds to the same active site as a known mTOR inhibitor (Figure 4A), demonstrating superior binding energy and inhibitory potential. Interaction profiling showed a key hydrogen bond with ASP950 and hydrophobic contacts with MET953, TRP812, ILE881, ILE963, ALA885, and LYS883 (Figure 4B), indicating high target specificity and structural stability. These findings suggest a potential mechanism of action for tiliacorinine against mTOR. Molecular dynamics simulations confirmed stable and specific binding of tiliacorinine to mTOR, particularly at ASP950, TRP1086, and PHE1087. These interactions, including hydrogen bonds and hydrophobic contacts, align with docking results and support tiliacorinine’s strong affinity and selectivity for the mTOR active site (Figure 9 and Figure 10). mTOR is a key regulator of cell growth, proliferation, and angiogenesis, making it a promising target for anticancer drug development. Rapamycin and its analogs have shown clinical success [75]. Emerging ATP-competitive inhibitors—both mTOR-selective (e.g., OSI-0279, INK-12810, CC-22311) and dual mTOR/PI3K agents (e.g., PF-04691502, BEZ235, GSK2126458)—are undergoing evaluation in clinical trials [76]. This study combines network pharmacology, molecular docking, and molecular dynamics simulation to elucidate tiliacorinine’s mechanism of action, highlighting its potential as an mTOR inhibitor. The findings lay a solid groundwork for future experimental and clinical research in CCA treatment. However, its mTOR-related anticancer effects require further validation through in vitro and in vivo studies.

## 5. Conclusions

This study integrates ADMET profiling, network pharmacology, molecular docking, and molecular dynamics simulations to elucidate the multi-targeted therapeutic potential of tiliacorinine in CCA. Functional enrichment analyses (GO and KEGG) revealed that key hub genes are involved in cell migration, membrane structure, protein kinase activity, and cancer-related pathways, including central carbon metabolism and microRNA regulation. Network and PPI analyses identified SRC, HIF1A, HSP90AA1, NFKB1, MTOR, MMP9, MMP2, PIK3CA, ICAM1, and MAPK1 as core targets. Tiliacorinine showed strong binding affinities to SRC, MMP9, MAPK1, and especially mTOR, with the highest docking score and stable interactions confirmed by MD simulations at ASP950, TRP1086, and PHE1087. These findings provide a promising foundation for developing novel CCA therapeutics, though experimental validation in vitro and in vivo remains essential.

## Figures and Tables

**Table 1:** Predicted Physicochemical and Drug-Likeness Profiles of Tiliacorinine via SWISS Analysis.

**Physicochemical Properties**	
Formula	C_36_H_36_N_2_O_5_
Molecular weight	576.68 g/mol
Num. heavy atoms	43
Num. arom. heavy atoms	24
Fraction Csp3	0.33
Num. rotatable bonds	2
Num. H-bond acceptors	7
Num. H-bond donors	1
Molar Refractivity	174.11
TPSA	63.63 Å^2^
**Lipophilicity**	
Log Po/w (iLOGP)	4.96
Log Po/w (XLOGP3)	6.07
Log Po/w (WLOGP)	5.42
Log Po/w (MLOGP)	3.89
Log Po/w (SILICOS-IT)	5.85
Consensus Log Po/w	5.24
**Water Solubility**	
Log S (ESOL)	−7.52
Solubility	1.74 × 10^−5^ mg/mL; 3.02 × 10^−8^ mol/L
Class	Poorly soluble
Log S (Ali)	−7.19
Solubility	3.76 × 10^−5^ mg/mL; 6.51 × 10^−8^ mol/L
Class	Poorly soluble
Log S (SILICOS-IT)	−10.03
Solubility	5.41 × 10^−8^ mg/mL; 9.38 × 10^−11^ mol/L
Class	Insoluble
**Drug-likeness**	
Lipinski	Yes; 1 violation: MW > 500
Ghose	No; 3 violations: MW > 480, MR > 130, #atoms > 70
Veber	Yes
Egan	Yes
Muegge	No; 2 violations: XLOGP3 > 5, #rings > 7
Bioavailability Score	0.55

**Table 2:** Predicted Absorption, Distribution, Metabolism, Excretion, and Toxicity (ADMET) of Tiliacorinine by pKCSM Computational Model.

Property	Model Name	Predicted Value	Unit
**Absorption**	Water solubility	−3.62	Numeric (log mol/L)
	Caco2 permeability	0.63	Numeric (log Papp in 10^−6^ cm/s)
	Intestinal absorption (human)	93.558	Numeric (% Absorbed)
	Skin Permeability	−2.735	Numeric (log Kp)
	P-glycoprotein substrate	Yes	Categorical (Yes/No)
	P-glycoprotein I inhibitor	Yes	Categorical (Yes/No)
	P-glycoprotein II inhibitor	Yes	Categorical (Yes/No)
**Distribution**	VDss (human)	−1.152	Numeric (log L/kg)
	Fraction unbound (human)	0.287	Numeric (Fu)
	BBB permeability	−0.545	Numeric (log BB)
	CNS permeability	−1.198	Numeric (log PS)
**Metabolism**	CYP2D6 substrate	No	Categorical (Yes/No)
	CYP3A4 substrate	Yes	Categorical (Yes/No)
	CYP1A2 inhibitor	No	Categorical (Yes/No)
	CYP2C19 inhibitor	Yes	Categorical (Yes/No)
	CYP2C9 inhibitor	No	Categorical (Yes/No)
	CYP2D6 inhibitor	No	Categorical (Yes/No)
	CYP3A4 inhibitor	No	Categorical (Yes/No)
**Excretion**	Total Clearance	0.762	Numeric (log mL/min/kg)
	Renal OCT2 substrate	No	Categorical (Yes/No)
**Toxicity**	AMES toxicity	Yes	Categorical (Yes/No)
	Max. tolerated dose (human)	0.244	Numeric (log mg/kg/day)
	hERG I inhibitor	No	Categorical (Yes/No)
	hERG II inhibitor	Yes	Categorical (Yes/No)
	Oral Rat Acute Toxicity (LD_50_)	2.527	Numeric (mol/kg)
	Oral Rat Chronic Toxicity (LOAEL)	0.513	Numeric (log mg/kg_bw/day)
	Hepatotoxicity	No	Categorical (Yes/No)
	Skin Sensitization	No	Categorical (Yes/No)
	*T. pyriformis* toxicity	0.285	Numeric (log ug/L)
	Minnow toxicity	1.623	Numeric (log mM)

**Table 3:** Computational Docking Evaluation of Tiliacorinine Against Molecular Targets in Cholangiocarcinoma (CCA).

No.	Protein Name	PDB	Compound and Positive Control	Binding Energies (kcal/mol)	Inhibition Constant (nM)	Hydrogen Bond Interactions
1	SRC	1Y57	Tiliacorinine	−9.29	155.79	GLU310, MET341, ASP348, ASN391
			MPZ600	−8.77	369.78	ALA293, ILE336, GLU310, MET341, ASP348
2	HIF1A	3KCX	Tiliacorinine	−7.32	4310.00	TYR102
			CQL	−6.67	12,960.00	HIS199, ASP201
3	HSP90AA1	4AWQ	Tiliacorinine	−7.38	3910.00	-
			5921224	−13.91	0.06414	GLN23, ASP93, MET98, ILE104
4	NFKB1	8TQD	Tiliacorinine	−6.09	34,330.00	HIS143, VAL144, THR145
			Dexamethasone	−6.99	7500.00	THR126, GLY128, ASP131, VAL133, GLY135, ALA137
5	MTOR	3TL5	Tiliacorinine	−10.78	12.62	ALA885, ASP950
			GDC-0980	−10.08	41.14	LYS802, VAL803, ALA805, ASP836, ASP841
6	MMP9	1GKC	Tiliacorinine	−9.42	125.43	GLU111, ALA189, ALA191
			NFH1448	−7.95	1490.00	GLY186, LEU188, ALA189, GLU402, TYR423
7	MMP2	8H78	Tiliacorinine	−6.72	1183.00	ASP77, LEU83, ALA86, VAL118
			L2U207	−11.61	3.07	TYR74, LEU83, ALA84, HIS85, VAL118, ILE142, THR144
8	PIK3CA	4JPS	Tiliacorinine	−8.99	259.27	GLN589, ASP933
			1LT1102	−10.31	27.69	LYS802, VAL851, SER854, GLN859
9	ICAM1	1IAM	Tiliacorinine	−8.00	1360.00	ILE10, GLU53
			BKA99414	−6.64	1365.00	LEU94, GLN181
10	MAPK1	6SLG	Tiliacorinine	−9.30	151.80	LYS151, SER153, CYS166
			LHZ401	−8.23	923.74	LYS54, MET108, ASP106, ASP111, ASN154, CYS166, ASP167

## Data Availability

Data is contained within the article and supplementary material.

## Supplementary Materials

The following supporting information can be downloaded at: https://www.mdpi.com/article/10.3390/antiox14111273/s1, Figure S1: The molecular dynamics simulation of tiliacorinine and GDC-0980 with MTOR over a 200 ns trajectory, with RMSF profiles in panels A and B illustrating residue flexibility at viable ligand contact sites for tiliacorinine–MTOR and GDC-0980–MTOR complexes, respectively; Figure S2: Molecular dynamics simulations of tiliacorinine and GDC-0980 bound to MTOR over a 200 ns trajectory; Table S1: Predicted Pharmacokinetics and Toxicity of Tiliacorinine by ProTox-3.0 and admetSAR Computational Model; Table S2: Details of the Protein targets in the PDB database and the Grid docking Parameters in Molecular docking.

## Abbreviations

The following abbreviations are used in this manuscript: Cholangiocarcinoma (CCA); Molecular dynamics (MD), Root Mean Square Deviation (RMSD); Root Mean Square Fluctuation (RMSF); SRC Proto-Oncogene (SRC); Hypoxia Inducible Factor 1 Subunit Alpha (HIF1A); Heat Shock Protein 90 Alpha Family Class A Member 1 (HSP90AA1); Nuclear Factor Kappa B Subunit 1 (NFKB1); Mechanistic Target of Rapamycin Kinase (MTOR); Matrix Metallopeptidase 9 (MMP9); Matrix Metallopeptidase 2 (MMP2); Phosphatidylinositol-4,5-Bisphosphate 3-Kinase Catalytic Subunit Alpha (PIK3CA); Intercellular Adhesion Molecule 1 (ICAM1) and Mitogen-Activated Protein Kinase 1 (MAPK1).

## References

[1] Bray F., Laversanne M., Sung H., Ferlay J., Siegel R.L., Soerjomataram I., Jemal A. (2024). Global cancer statistics 2022: GLOBOCAN estimates of incidence and mortality worldwide for 36 cancers in 185 countries. CA Cancer J. Clin..

[2] Banales J.M., Marin J.J.G., Lamarca A., Rodrigues P.M., Khan S.A., Roberts L.R., Cardinale V., Carpino G., Andersen J.B., Braconi C. (2020). Cholangiocarcinoma 2020: The next horizon in mechanisms and management. Nat. Rev. Gastroenterol. Hepatol..

[3] Kirstein M.M., Vogel A. (2016). Epidemiology and risk factors of cholangiocarcinoma. Visc. Med..

[4] Bergquist A., von Seth E. (2015). Epidemiology of cholangiocarcinoma. Best Pract. Res. Clin. Gastroenterol..

[5] Plentz R.R., Malek N.P. (2015). Clinical presentation, risk factors and staging systems of cholangiocarcinoma. Best Pract. Res. Clin. Gastroenterol..

[6] Bertuccio P., Malvezzi M., Carioli G., Hashim D., Boffetta P., El-Serag H.B., La Vecchia C., Negri E. (2019). Global trends in mortality from intrahepatic and extrahepatic cholangiocarcinoma. J. Hepatol..

[7] Sripa B., Pairojkul C. (2008). Cholangiocarcinoma: Lessons from Thailand. Curr. Opin. Gastroenterol..

[8] Khan A., Dageforde L. (2019). Cholangiocarcinoma. Surg. Oncol. Clin. N. Am..

[9] Alsaleh M., Leftley Z., Barbera T.A., Sithithaworn P., Khuntikeo N., Loilome W., Yongvanit P., Cox I.J., Chamodol N., Syms R.R.A. (2019). Cholangiocarcinoma: A guide for the nonspecialist. Int. J. Gen. Med..

[10] Wongjarupong N., Assavapongpaiboon B., Susantitaphong P., Cheungpasitporn W., Treeprasertsuk S., Rerknimitr R., Chaiteerakij R. (2017). Non-alcoholic fatty liver disease as a risk factor for cholangiocarcinoma: A systematic review and meta-analysis. BMC Gastroenterol..

[11] Sripa B., Bethony J.M., Sithithaworn P., Kaewkes S., Mairiang E., Loukas A., Mulvenna J., Laha T., Hotez P.J., Brindley P.J. (2011). Opisthorchiasis and Opisthorchis-associated cholangiocarcinoma in Thailand and Laos. Acta Trop..

[12] Perakanya P., Ungcharoen R., Worrabannakorn S., Ongarj P., Artchayasawat A., Boonmars T., Boueroy P. (2022). Prevalence and risk factors of Opisthorchis viverrini infection in Sakon Nakhon Province, Thailand. Trop. Med. Infect. Dis..

[13] Labib P.L., Goodchild G., Pereira S.P. (2019). Molecular pathogenesis of cholangiocarcinoma. BMC Cancer.

[14] Esmail A., Badheeb M., Alnahar B.W., Almiqlash B., Sakr Y., Al-Najjar E., Awas A., Alsayed M., Khasawneh B., Alkhulaifawi M. (2024). The recent trends of systemic treatments and locoregional therapies for cholangiocarcinoma. Pharmaceuticals.

[15] Nasim N., Sandeep I.S., Mohanty S. (2022). Plant-derived natural products for drug discovery: Current approaches and prospects. Nucleus.

[16] Das G., Gouda S., Kerry R.G., Cortes H., Prado-Audelo M.L.D., Leyva-Gómez G., Tsouh Fokou P.V., Gutiérrez-Grijalva E.P., Heredia J.B., Shin H.-S. (2022). Study of Traditional Uses, Extraction Procedures, Phytochemical Constituents, and Pharmacological Properties of *Tiliacora Triandra*. J. Chem..

[17] Nutmakul T. (2021). Phytochemical and pharmacological activity of *Tiliacora Triandra* (Colebr.) diels. Songklanakarin J. Sci. Technol..

[18] Lumlerdkij N., Boonrak R., Booranasubkajorn S., Akarasereenont P., Heinrich M. (2020). In vitro protective effects of plants frequently used traditionally in cancer prevention in Thai traditional medicine: An ethnopharmacological study. J. Ethnopharmacol..

[19] Rattana S., Cushnie B., Taepongsorat L., Phadungkit M. (2015). Chemical constituents and in vitro anticancer activity of *Tiliacora triandra* leaves. Pharmacogn. J..

[20] Manosroi A., Akazawa H., Akihisa T., Jantrawut P., Kitdamrongtham W., Manosroi W., Manosroi J. (2015). In vitro anti-proliferative activity on colon cancer cell line (HT-29) of Thai medicinal plants selected from Thai/Lanna medicinal plant recipe database “MANOSROI III”. J. Ethnopharmacol..

[21] Jongrungraungchok S., Madaka F., Wunnakup T., Sudsai T., Pongphaew C., Songsak T., Pradubyat N. (2023). In vitro antioxidant, anti-inflammatory, and anticancer activities of mixture Thai medicinal plants. BMC Complement. Med. Ther..

[22] Janeklang S., Nakaew A., Vaeteewoottacharn K., Seubwai W., Boonsiri P., Kismali G., Suksamrarn A., Okada S., Wongkham S. (2014). In vitro and in vivo antitumor activity of tiliacorinine in human cholangiocarcinoma. Asian Pac. J. Cancer Prev..

[23] Wiriyachitra P., Phuriyakorn B. (1981). Alkaloids of *Tiliacora triandra*. Aust. J. Chem..

[24] Sureram S., Senadeera S.P.D., Hongmanee P., Mahidol C., Ruchirawat S., Kittakoop P. (2012). Antimycobacterial activity of bisbenzylisoquinoline alkaloids from *Tiliacora triandra* against multidrug-resistant isolates of *Mycobacterium tuberculosis*. Bioorganic Med. Chem. Lett..

[25] Pradubyat N., Madaka F., Songsak T., Jongrungruangchok S. (2024). In Vitro Biological Activity of *Tiliacora triandra* (Colebr.) Diels Root Extract. J. Curr. Sci. Technol..

[26] Detarya M., Mahalapbutr P., Waenphimai O., Kidoikhammouan S., Janeklang S., Sawanyawisuth K., Vaeteewoottacharn K., Seubwai W., Saengboonmee C., Thothaisong T. (2023). Induction of apoptotic cell death of cholangiocarcinoma cells by tiliacorinine from *Tiliacora triandra*: A mechanistic insight. Biochim. Et Biophys. Acta General. Subj..

[27] Yang M., Chen J.L., Xu L.W., Ji G. (2013). Navigating traditional Chinese medicine network pharmacology and computational tools. Evid. Based Complement. Alternat. Med..

[28] Hopkins A.L. (2007). Network Pharmacology. Nat. Biotechnol..

[29] İslamoğlu F. (2024). Molecular docking, bioactivity, ADME, toxicity risks, and quantum mechanical parameters of some 1,2-dihydroquinoline derivatives were calculated theoretically for investigation of their use as pharmaceutical active ingredients in the treatment of multiple sclerosis (MS). Prospect. Pharm. Sci..

[30] Zuo H., Zhang Q., Su S., Chen Q., Yang F., Hu Y. (2018). A network pharmacology-based approach to analyse potential targets of traditional herbal formulas: An example of Yu Ping Feng decoction. Sci. Rep..

[31] Macalino S.J.Y., Gosu V., Hong S., Choi S. (2015). Role of computer-aided drug design in modern drug discovery. Arch. Pharm. Res..

[32] Pinzi L., Rastelli G. (2019). Molecular Docking: Shifting Paradigms in Drug Discovery. Int. J. Mol. Sci..

[33] Daina A., Michielin O., Zoete V. (2017). SwissADME: A free web tool to evaluate pharmacokinetics, drug-likeness and medicinal chemistry friendliness of small molecules. Sci. Rep..

[34] Pires D.E.V., Blundell T.L., Ascher D.B. (2015). pkCSM: Predicting small-molecule pharmacokinetic properties using graph-based signatures. J. Med. Chem..

[35] Daina A., Michielin O., Zoete V. (2019). SwissTargetPrediction: Updated data and new features for efficient prediction of protein targets of small molecules. Nucleic Acids Res..

[36] Heberle H., Meirelles G.V., da Silva F.R., Telles G.P., Minghim R. (2015). InteractiVenn: A web-based tool for the analysis of sets through Venn diagrams. BMC Bioinform..

[37] Szklarczyk D., Kirsch R., Koutrouli M., Nastou K., Mehryary F., Hachilif R., Gable A.L., Fang T., Doncheva N.T., Pyysalo S. (2023). The STRING database in 2023: Protein–protein association networks and functional enrichment analyses for any sequenced genome of interest. Nucleic Acids Res..

[38] Ge S.X., Jung D., Yao R. (2019). ShinyGO: A graphical gene-set enrichment tool for animals and plants. Bioinformatics.

[39] Burley S.K., Bhikadiya C., Bi C., Bittrich S., Chen L., Crichlow G.V., Christie C.H., Dalenberg K., Di Costanzo L., Duarte J.M. (2021). RCSB Protein Data Bank: Powerful new tools for exploring 3D structures of biological macromolecules for basic and applied research and education. Nucleic Acids Res..

[40] Kim S., Chen J., Cheng T., Gindulyte A., He J., He S., Li Q., Shoemaker B.A., Thiessen P.A., Yu B. (2021). PubChem in 2021: New data content and improved web interfaces. Nucleic Acids Res..

[41] Pettersen E.F., Goddard T.D., Huang C.C., Couch G.S., Greenblatt D.M., Meng E.C., Ferrin T.E. (2004). UCSF Chimera—A visualization system for exploratory research and analysis. J. Comput. Chem..

[42] Morris G.M., Huey R., Lindstrom W., Sanner M.F., Belew R.K., Goodsell D.S., Olson A.J. (2009). AutoDock4 and AutoDockTools4: Automated docking with selective receptor flexibility. J. Comput. Chem..

[43] Sama-ae I., Pattaranggoon N.C., Tedasen A. (2023). In silico prediction of antifungal compounds from natural sources towards lanosterol 14-alpha demethylase (CYP51) using molecular docking and molecular dynamics simulation. J. Mol. Graph. Model..

[44] Tedasen A., Chiabchalard A., Tencomnao T., Yamasaki K., Majima H.J., Phongphithakchai A., Chatatikun M. (2024). Anti-melanogenic activity of ethanolic extract from Garcinia atroviridis fruits using in vitro experiments, network pharmacology, molecular docking, and molecular dynamics simulation. Antioxidants.

[45] Khan A., Tareen A., Ashraf S.U., Mufti I.S., Karam M., Ahmed F.I., Rasool S.N., Akhtar R., Amin A., Khan S.R. (2025). Intrahepatic cholangiocarcinoma mortality in the USA, 1999–2020: A 21-year population-based analysis. Cancer Causes Control.

[46] Rimassa L., Khan S., Groot Koerkamp B., Roessler S., Andersen J.B., Raggi C., Lleo A., Nault J.C., Calderaro J., Gabbi C. (2025). Mapping the landscape of biliary tract cancer in Europe: Challenges and controversies. Lancet Reg. Health Eur..

[47] Lei Z.N., Tian Q., Teng Q.X., Wurpel J.N.D., Zeng L., Pan Y., Chen Z.S. (2023). Understanding and targeting resistance mechanisms in cancer. MedComm..

[48] Pillai O., Dhanikula A.B., Panchagnula R. (2001). Drug delivery: An odyssey of 100 years. Curr. Opin. Chem. Biol..

[49] Rai M., Singh A.V., Paudel N., Kanase A., Falletta E., Kerkar P., Heyda J., Barghash R.F., Singh S.P., Soos M. (2023). Herbal concoction unveiled: A computational analysis of phytochemicals’ pharmacokinetic and toxicological profiles using novel approach methodologies (NAMs). Curr. Res. Toxicol..

[50] Ferreira L.L.G., Andricopulo A.D. (2019). ADMET modeling approaches in drug discovery. Drug Discov. Today.

[51] Montanari F., Kuhnke L., ter Laak A., Clevert D.-A. (2019). Modeling physico-chemical ADMET endpoints with multitask graph convolutional networks. Molecules.

[52] Pongchairerk U., Guan J.L., Leardkamolkarn V. (2005). Focal adhesion kinase and Src phosphorylations in HGF-induced proliferation and invasion of human cholangiocarcinoma cell line, HuCCA-1. World J. Gastroenterol..

[53] Wu W.-S., Ling C.-H., Lee M.-C., Cheng C.-C., Chen R.-F., Lin C.-F., You R.-I., Chen Y.-C. (2022). Targeting Src-Hic-5 signal cascade for preventing migration of cholangiocarcinoma cell HuCCT1. Biomedicines.

[54] Dash S., Hanson S., King B., Nyswaner K., Foss K., Tesi N., Harvey M.J.B., Navarro-Marchal S.A., Woods A., Poradosu E. (2024). The SRC family kinase inhibitor NXP900 demonstrates potent antitumor activity in squamous cell carcinomas. J. Biol. Chem..

[55] Yu A., Zhao L., Kang Q., Li J., Chen K., Fu H. (2020). Transcription factor HIF1α promotes proliferation, migration, and invasion of cholangiocarcinoma via long noncoding RNA H19/microRNA-612/Bcl-2 axis. Transl. Res. J. Lab. Clin. Med..

[56] Thongchot S., Yongvanit P., Loilome W., Seubwai W., Phunicom K., Tassaneeyakul W., Namwat N. (2014). High expression of HIF-1α, BNIP3 and PI3KC3: Hypoxia-induced autophagy predicts cholangiocarcinoma survival and metastasis. Asian Pac. J. Cancer Prev..

[57] Shirota T., Ojima H., Hiraoka N., Shimada K., Rokutan H., Arai Y., Kanai Y., Miyagawa S., Shibata T. (2015). Heat shock protein 90 is a potential therapeutic target in cholangiocarcinoma. Mol. Cancer Ther..

[58] Yan L.R., Shen S.X., Wang A., Ding H.X., Liu Y.N., Yuan Y., Xu Q. (2021). Comprehensive pan-cancer analysis of heat shock protein 110, 90, 70, and 60 families. Front. Mol. Biosci..

[59] Shahana M.V., Choudhary B.H. (2025). HSP90 and the cancer transcriptome: A comprehensive review of inhibitors and mechanistic insights. Int. J. Clin. Oncol..

[60] Seubwai W., Wongkham C., Puapairoj A., Khuntikeo N., Pugkhem A., Hahnvajanawong C., Chaiyagool J., Umezawa K., Okada S., Wongkham S. (2014). Aberrant expression of NF-κB in liver fluke associated cholangiocarcinoma: Implications for targeted therapy. PLoS ONE.

[61] Song Z., Feng Z., Wang X., Li J., Zhang D. (2025). NFKB1 as a key player in tumor biology: From mechanisms to therapeutic implications. Cell Biol. Toxicol..

[62] Daugan C., Boidot R., Ghiringhelli F., Borg C., Vienot A. (2024). Targeting mTOR signaling for the treatment of intrahepatic cholangiocarcinoma with TSC1/ARID1A mutations: A case report with an unexpected response. Ther. Adv. Med. Oncol..

[63] Wu C.-E., Chen M.-H., Yeh C.-N. (2019). mTOR inhibitors in advanced biliary tract cancers. Int. J. Mol. Sci..

[64] Pan S., Hu Y., Hu M., Jian H., Chen M., Gan L., Zheng P., He Y., Wang J. (2020). Platelet-derived PDGF promotes the invasion and metastasis of cholangiocarcinoma by upregulating MMP2/MMP9 expression and inducing EMT via the p38/MAPK signaling pathway. Am. J. Transl. Res..

[65] Joechle K., Jumaa H., Thriene K., Hellerbrand C., Kulemann B., Fichtner-Feigl S., Lang S.A., Guenzle J. (2022). Dual inhibition of mTORC1/2 reduces migration of cholangiocarcinoma cells by regulation of matrix metalloproteinases. Front. Cell Dev. Biol..

[66] Ballin N., Ott A., Seibel-Kelemen O., Bonzheim I., Nann D., Beha J., Spahn S., Singer S., Ossowski S., Roggia C. (2025). Case report: FGFR2 inhibitor resistance via PIK3CA and CDKN2A/B in an intrahepatic cholangiocarcinoma patient with FGFR2-SH3GLB1 fusion. Front. Oncol..

[67] Deshpande V., Nduaguba A., Zimmerman S.M., Kehoe S.M., MacConaill L.E., Lauwers G.Y., Ferrone C., Bardeesy N., Zhu A.X., Hezel A.F. (2011). Mutational profiling reveals PIK3CA mutations in gallbladder carcinoma. BMC Cancer.

[68] Janan M., Proungvitaya S., Limpaiboon T., Proungvitaya T., Roytrakul S., Wongkham C., Jearanaikoon P., Chur-in S., Wongkham S. (2012). Serum adhesion molecule-1 (ICAM-1) as a potential prognostic marker for cholangiocarcinoma patients. Asian Pac. J. Cancer Prev..

[69] Zhu B., Wang X., Shimura T., Huang A.C., Kong N., Dai Y., Fang J., Guo P., Ying J.E. (2023). Development of potent antibody drug conjugates against ICAM1⁺ cancer cells in preclinical models of cholangiocarcinoma. NPJ Precis. Oncol..

[70] Zheng Y., Zhou C., Yu X.X., Wu C., Jia H.L., Gao X.M., Yang J.M., Wang C.Q., Luo Q., Zhu Y. (2018). Osteopontin promotes metastasis of intrahepatic cholangiocarcinoma through recruiting MAPK1 and mediating Ser675 phosphorylation of β-catenin. Cell Death Dis..

[71] Chen C., Nelson L.J., Ávila M.A., Cubero F.J. (2019). Mitogen-Activated Protein Kinases (MAPKs) and Cholangiocarcinoma: The Missing Link. Cells.

[72] Landskron G., De la Fuente M., Thuwajit P., Thuwajit C., Hermoso A.M. (2014). Chronic inflammation and cytokines in the tumor microenvironment. J. Immunol. Res..

[73] Caligiuri A., Becatti M., Porro N., Borghi S., Marra F., Pastore M., Taddei N., Fiorillo C., Gentilini A. (2024). Oxidative Stress and Redox-Dependent Pathways in Cholangiocarcinoma. Antioxidants.

[74] Wu Z. (2024). Transcriptomic analysis reveals oxidative stress-related signature and molecular subtypes in cholangio carcinoma. Mol. Genet. Genom..

[75] Abraham R.T., Eng C.H. (2008). Mammalian target of rapamycin as a therapeutic target in oncology. Expert. Opin. Ther. Targets.

[76] Wang L., Chen L., Yu M., Xu L.H., Cheng B., Lin Y.S., Gu Q., He X.H., Xu J. (2016). Discovering new mTOR inhibitors for cancer treatment through virtual screening methods and in vitro assays. Sci. Rep..

