# Supplementary material for: Tiliacorinine as a Promising Candidate for Cholangiocarcinoma Therapy via Oxidative Stress Molecule Modulation: A Study Integrating Network Pharmacology, Molecular Docking and Molecular Dynamics Simulation
Source: Antioxidants (Basel). 2025 Oct 23;14(11):1273. doi: 10.3390/antiox14111273 (PMC12649441; doi:10.3390/antiox14111273)
Supplement: Supplementary file 1 [file antioxidants-14-01273-s001.zip › antioxidants-3873728-supplementary.pdf]

Supplement Table S1. Predicted Pharmacokinetics and Toxicity of Tiliacorinine by ProTox-3.0 and admetSAR Computational Model.

**Prediction of TOXicity of chemicals by ProTox-3.0**			
**Classification**	**Target**	**Prediction**	**Probability**
Organ toxicity	Hepatotoxicity	Inactive	0.95
	Neurotoxicity	Active	0.60
	Nephrotoxicity	Inactive	0.59
	Respiratory toxicity	Active	0.93
	Cardiotoxicity	Inactive	0.78
Toxicity end points	Carcinogenicity	Inactive	0.55
	Immunotoxicity	Active	0.99
	Mutagenicity	Active	0.77
	Cytotoxicity	Inactive	0.62
	BBB-barrier	Active	0.86
	Ecotoxicity	Inactive	0.52
	Clinical toxicity	Active	0.50
	Nutritional toxicity	Active	0.51
Tox21-Nuclear receptor signalling pathways	Aryl hydrocarbon Receptor (AhR)	Inactive	0.69
	Androgen Receptor (AR)	Inactive	0.88
	Androgen Receptor Ligand Binding Domain (AR-LBD)	Inactive	0.99
	Aromatase	Inactive	0.90
	Estrogen Receptor Alpha (ER)	Inactive	0.95
	Estrogen Receptor Ligand Binding Domain (ER-LBD)	Inactive	0.99
	Peroxisome Proliferator Activated Receptor Gamma (PPAR-Gamma)	Inactive	0.99
Tox21-Stress response pathways	Nuclear factor (erythroid-derived 2)-like 2/antioxidant responsive element (nrf2/ARE)	Inactive	0.98
	Heat shock factor response element (HSE)	Inactive	0.98
	Mitochondrial Membrane Potential (MMP)	Inactive	0.89
	Phosphoprotein (Tumor Supressor) p53	Inactive	0.96
	ATPase family AAA domain-containing protein 5 (ATAD5)	Inactive	0.99
Molecular Initiating Events	Thyroid hormone receptor alpha (THRα)	Inactive	0.82
	Thyroid hormone receptor beta (THRβ)	Inactive	0.81
	Transtyretrin (TTR)	Inactive	0.93
	Ryanodine receptor (RYR)	Inactive	0.67
	GABA receptor (GABAR)	Inactive	0.67
	Glutamate N-methyl-D-aspartate receptor (NMDAR)	Inactive	0.66
	alpha-amino-3-hydroxy-5-methyl-4-isoxazolepropionate receptor (AMPAR)	Inactive	0.99
	Kainate receptor (KAR)	Inactive	1.00
	Achetylcholinesterase (AChE)	Active	0.57
	Constitutive androstane receptor (CAR)	Inactive	1.00
	Pregnane X receptor (PXR)	Inactive	0.59
	NADH-quinone oxidoreductase (NADHOX)	Inactive	0.82
	Voltage gated sodium channel (VGSC)	Inactive	0.75

	Na+/I- symporter (NIS)	Inactive	0.91
Metabolism	Cytochrome CYP1A2	Inactive	0.94
	Cytochrome CYP2C19	Inactive	0.97
	Cytochrome CYP2C9	Inactive	0.80
	Cytochrome CYP2D6	Active	0.66
	Cytochrome CYP3A4	Inactive	0.94
	Cytochrome CYP2E1	Inactive	0.99

ADMET Predicted Profile by admetSAR

**Classification**	**Target**	**Prediction**	**Probability**
Absorption	Blood-Brain Barrier	BBB+	0.9782
	Human Intestinal Absorption	HIA+	0.9266
	Caco-2 Permeability	Caco2+	0.8496
	P-glycoprotein Substrate	Substrate	0.8431
	P-glycoprotein Inhibitor	Inhibitor	0.5621
		Non-inhibitor	0.8940
	Renal Organic Cation Transporter	Inhibitor	0.5088
Distribution	Subcellular localization	Mitochondria	0.4491
Metabolism	CYP450 2C9 Substrate	Non-substrate	0.8270
	CYP450 2D6 Substrate	Non-substrate	0.5000
	CYP450 3A4 Substrate	Substrate	0.5958
	CYP450 1A2 Inhibitor	Non-inhibitor	0.8931
	CYP450 2C9 Inhibitor	Non-inhibitor	0.9575
	CYP450 2D6 Inhibitor	Non-inhibitor	0.9048
	CYP450 2C19 Inhibitor	Non-inhibitor	0.9382
	CYP450 3A4 Inhibitor	Non-inhibitor	0.9342
	CYP Inhibitory Promiscuity	Low CYP Inhibitory Promiscuity	0.9726
Toxicity	Human Ether-a-go-go-Related Gene Inhibition (hERG)	Weak inhibitor	0.7513
		Non-inhibitor	0.6207
	AMES Toxicity	AMES toxic	0.8664
	Carcinogens	Non-carcinogens	0.9451
	Fish Toxicity	High FHMT	0.8124
	Tetrahymena Pyriformis Toxicity	High TPT	0.9021
	Honey Bee Toxicity	Low HBT	0.6272
	Biodegradation	Not ready biodegradable	0.9851
	Acute Oral Toxicity	III	0.7725
	Carcinogenicity (Three-class)	Non-required	0.6538

Supplement Table S2. Details of the Protein targets in the PDB database and the Grid docking Parameters in Molecular docking.

**Targets**	**PDB ID**	**Method**	**Co-ligand / drug**	**Ligand full name**	**Resolution (Å)**	**R-Value Free**	**R-Value Work**	**Spacing (Å)**	**Grid box size (in xyz)**	**Center Grid Box**		
										**X Center**	**Y Center**	**Z Center**
SRC	1Y57	X-ray diffraction	MPZ600	4-[(4-methylpiperazin-1-yl)methyl]-N-{3-[(4-pyridin-3-ylpyrimidin-2-yl)amino]phenyl}benzamide (Co-crystal ligand)	1.91 Å	0.213	0.188	0.375	90 x 90 x 90	13.665	34.752	38.334
HIF1A	3KCX	X-ray diffraction	CQL	Clioquinol or 5-chloro-7-iodoquinolin-8-ol (Co-crystal ligand)	2.60 Å	0.292	0.244	0.375	60 x 60 x 60	-21.658	27.512	8.647
HSP90AA1	4AWQ	X-ray diffraction	5921224	N-benzyl-6-[(3-endo)-3-{[(3-methoxy-2-methylphenyl)carbonyl]amino}-8-azabicyclo[3.2.1]oct-8-yl]pyridine-3-carboxamide (Co-crystal ligand)	1.60 Å	0.262	0.227	0.375	70 x 70 x 70	-3.145	3.523	-5.547
NFKB1	8TQD	X-ray diffraction	Dexamethasone	Dexamethasone (Literature selected ligand from https://doi.org/10.1038/s41392-024-01757-9)	2.02 Å	0.227	0.182	0.375	60 x 60 x 60	7.871	-14.327	-12.488
MTOR	3TL5	X-ray diffraction	GDC-0980	(2S)-1-(4-{[2-(2-aminopyrimidin-5-yl)-7-methyl-4-(morpholin-4-yl)thieno[3,2-d]pyrimidin-6-yl]methyl}piperazin-1-yl)-2-hydroxypropan-1-one (Co-crystal ligand)	2.79 Å	0.267	0.199	0.375	60 x 60 x 60	19.377	62.831	20.179
MMP9	1GKC	X-ray diffraction	NFH1448	N~2~-[(2R)-2-{[formyl(hydroxy)amino]methyl}-4-methylpentanoyl]-N,3-dimethyl-L-valinamide (Co-crystal ligand)	2.30 Å	0.239	0.207	0.375	60 x 60 x 60	64.641	29.597	117.864
MMP2	8H78	X-ray diffraction	L2U207	(2~{R})-2-[[4-[(4-aminocarbonylphenyl)carbonylamino]phenyl]sulfonylamino]-5-[(2~{S},4~{S})-4-azanyl-2-[[(2~{S})-1-[[(2~{S})-1-[(5-azanyl-5-oxidanylidene-pentyl)amino]-5-oxidanyl-1,5-bis(oxidanylidene)pentan-2-yl]-methyl-amino]-4-methyl-1-oxidanylidene-pentan-2-yl]carbamoyl]pyrrolidin-1-yl]-5-oxidanylidene-pentanoic acid (Co-crystal ligand)	2.40 Å	0.319	0.265	0.375	75 x 75 x 75	25.843	24.565	-6.742
PIK3CA	4JPS	X-ray diffraction	1LT1102	(2S)-N~1~-{4-methyl-5-[2-(1,1,1-trifluoro-2-methylpropan-2-yl)pyridin-4-yl]-1,3-thiazol-2-yl}pyrrolidine-1,2-dicarboxamide (Co-crystal ligand)	2.20 Å	0.228	0.205	0.375	60 x 60 x 60	-2.909	-10.458	16.126
ICAM1	1IAM	X-ray diffraction	BKA99414 (PubChem CID10361323)	CAM-IN-1 or 4-(4-bromophenoxy)-N-methylthieno[2,3-c]pyridine-2-carboxamide (Literature selected ligand from https://doi.org/10.1021/jm0101702)	2.10 Å	0.303	0.214	0.375	126 x 116 x 92	33.99	80.762	0.082
MAPK1	6SLG	X-ray diffraction	LHZ401	(6~{R})-7-[[3,4-bis(fluoranyl)phenyl]methyl]-6-(methoxymethyl)-2-[5-methyl-2-[(2-methylpyrazol-3-yl)amino]pyrimidin-4-yl]-5,6-dihydroimidazo[1,2-a]pyrazin-8-one (Co-crystal ligand)	1.33 Å	0.228	0.206	0.375	60 x 60 x 60	-4.61	5.457	12.548

A

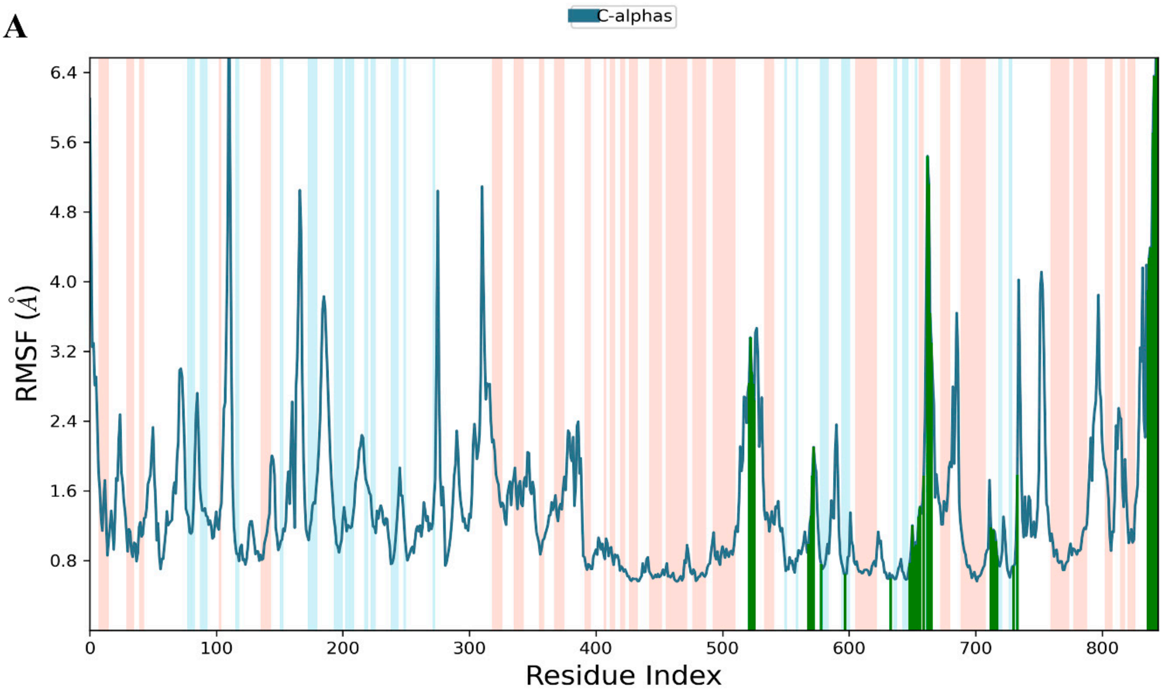

B

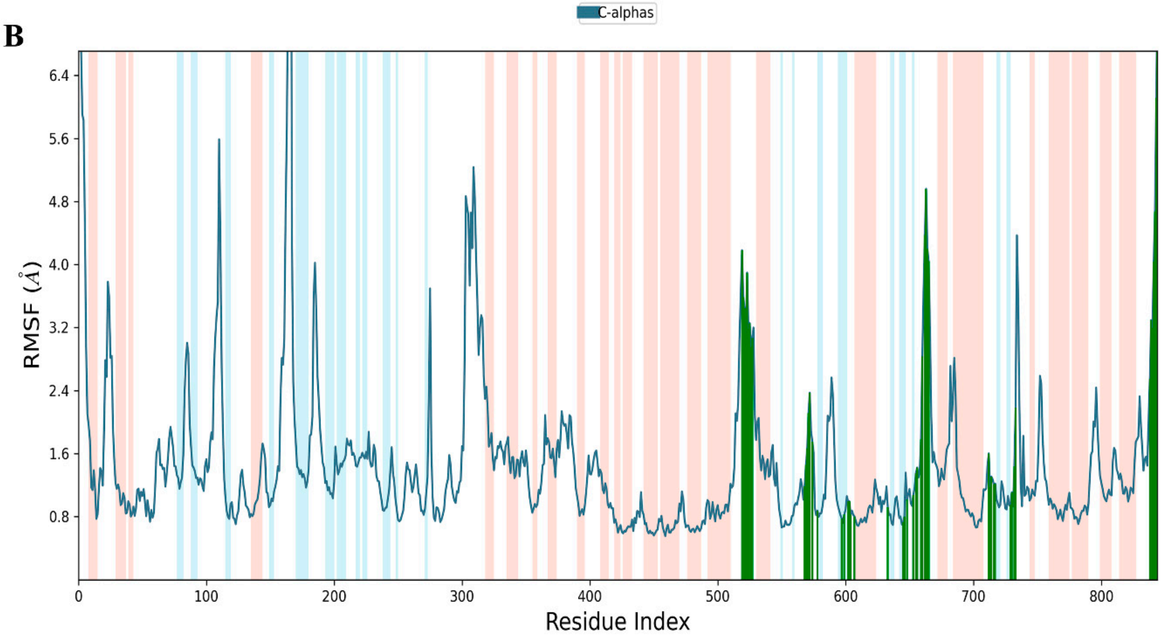

Supplement Figure S1. The molecular dynamics simulation of tiliacorinine and GDC-0980 with MTOR over a 200 ns trajectory, with RMSF profiles in panels A and B illustrating residue flexibility at viable ligand contact sites for tiliacorinine–MTOR and GDC-0980–MTOR complexes, respectively.

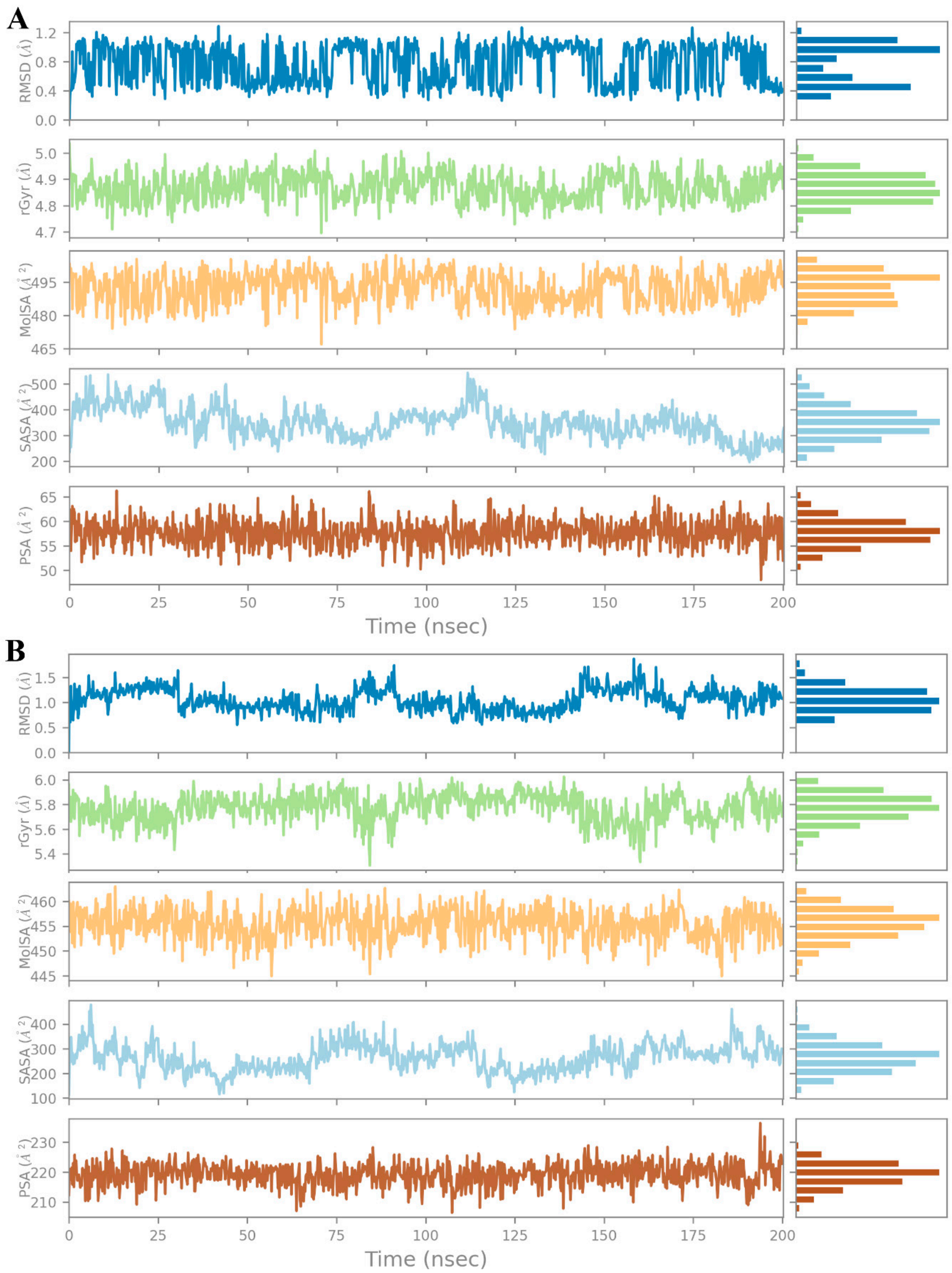

Supplement Figure S2. Molecular dynamics simulations of tiliacorinine and GDC-0980 bound to MTOR over a 200 ns trajectory. Panels A and B present the RMSD, rGyr, MolSA, SASA, and PSA profiles, highlighting residue flexibility at key ligand–MTOR contact sites for the tiliacorinine–MTOR and GDC-0980–MTOR complexes, respectively.
